# Multinational Observational Cohort Study of COVID-19–Associated Pulmonary Aspergillosis^1^

**DOI:** 10.3201/eid2711.211174

**Published:** 2021-11

**Authors:** Nico A.F. Janssen, Rémy Nyga, Lore Vanderbeke, Cato Jacobs, Mehmet Ergün, Jochem B. Buil, Karin van Dijk, Josje Altenburg, Catherine S.C. Bouman, Hans I. van der Spoel, Bart J.A. Rijnders, Albert Dunbar, Jeroen A. Schouten, Katrien Lagrou, Marc Bourgeois, Marijke Reynders, Niels van Regenmortel, Lynn Rutsaert, Piet Lormans, Simon Feys, Yves Debavaye, Fabienne Tamion, Damien Costa, Julien Maizel, Hervé Dupont, Taieb Chouaki, Saad Nseir, Boualem Sendid, Roger J.M. Brüggemann, Frank L. van de Veerdonk, Joost Wauters, Paul E. Verweij

**Affiliations:** Radboud University Medical Center, Nijmegen, the Netherlands (N.A.F Janssen, M. Ergün, J.B. Buil, J.A. Schouten, R.J.M. Brüggemann, F.L. van de Veerdonk, P.E. Verweij);; Amiens University Hospital, Amiens, France (R. Nyga, J. Maizel, H. Dupont, T. Chouaki);; University Hospitals Leuven, Leuven, Belgium (L. Vanderbeke, C. Jacobs, K. Lagrou, Y. Debavaye, J. Wauters);; Katholieke Universiteit Leuven, Leuven (L. Vanderbeke, K. Lagrou, J. Wauters);; Amsterdam University Medical Centers, Amsterdam, the Netherlands (K. van Dijk, J. Altenburg, C.S.C. Bouman, H. van der Spoel);; Erasmus Medical Center, Rotterdam, the Netherlands (B.J.A. Rijnders, A. Dunbar);; AZ Sint-Jan Brugge-Oostende, Brugge, Belgium (M. Bourgeois, M. Reynders);; ZNA Campus Stuivenberg, Antwerpen, Belgium (N. van Regenmortel, L. Rutsaert);; AZ Delta Hospital, Roeselare, Belgium (P. Lormans, S. Feys);; Rouen University Hospital, Rouen, France (F. Tamion, D. Costa);; Lille University Hospital, Lille, France (S. Nseir, B. Sendid);; University of Lille, Lille (S. Nseir, B. Sendid)

**Keywords:** respiratory infections, severe acute respiratory syndrome coronavirus 2, SARS-CoV-2, SARS, COVID-19, coronavirus disease, zoonoses, viruses, coronavirus, invasive pulmonary aspergillosis, mycology, epidemiology, risk factors, mortality rates, fungi, Aspergillus

## Abstract

We performed an observational study to investigate intensive care unit incidence, risk factors, and outcomes of coronavirus disease–associated pulmonary aspergillosis (CAPA). We found 10%–15% CAPA incidence among 823 patients in 2 cohorts. Several factors were independently associated with CAPA in 1 cohort and mortality rates were 43%–52%.

Incidence of coronavirus disease (COVID-19)–associated pulmonary aspergillosis (CAPA) in hospital intensive care units (ICUs) is 3.8%–33.3% (*1*–*9*). Variations might be explained by differences in patient populations and CAPA definitions used, complicating direct comparisons between studies.

Diagnosing CAPA is complex because cases frequently lack typical radiologic features and European Organization for Research and Treatment of Cancer and the Mycoses Study Group Education and Research Consortium (EORTC/MSGERC) host factors (*10*) and because mycologic evidence is difficult to obtain. Serum galactomannan (GM) detection has low sensitivity in CAPA (*7*,*10*). 

The European Confederation of Medical Mycology and International Society for Human and Animal Mycology (ECMM/ISHAM) published consensus criteria for a CAPA definition (*11*). We used these criteria to perform an observational cohort study to assess CAPA incidence in patients with COVID-19 admitted to ICUs during the first wave of the COVID-19 pandemic.

## The Study

We collected partially prospective and partially retrospective data for 823 patients in 2 cohorts. The discovery cohort comprised patients with PCR-confirmed or clinically presumed COVID-19 admitted to 4 ICUs in the Netherlands and 4 ICUs in Belgium during February 28–May 27, 2020. The validation cohort comprised patients with PCR-confirmed COVID-19 admitted because of respiratory insufficiency to 3 participating ICUs in France during April 7–May 31, 2020 (Appendix Methods, Table 1).

**Figure 1:**
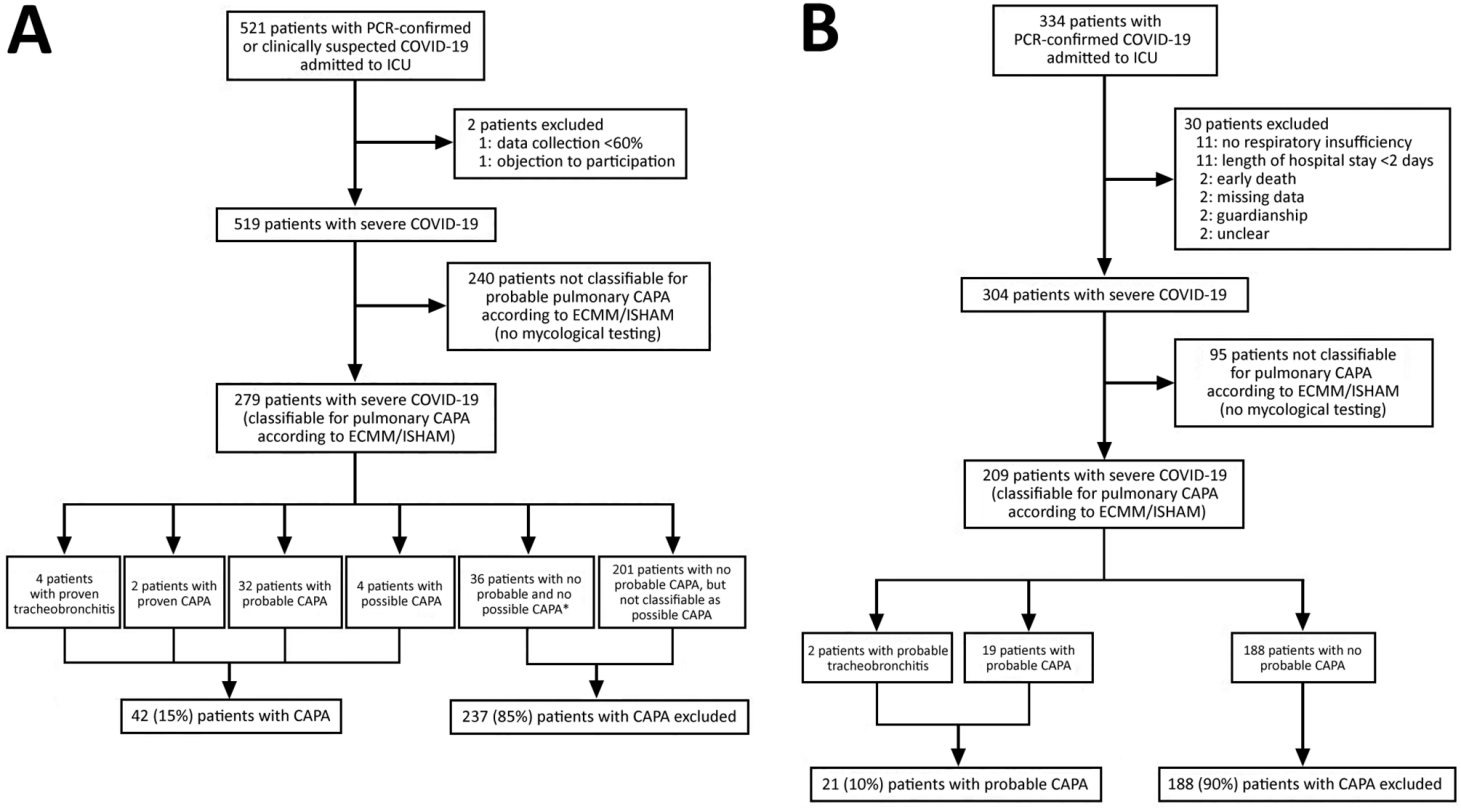
Flowchart of the study inclusion process for a multinational observational study of CAPA in 3 countries in Europe, 2020. A) Discovery cohort; B) validation cohort. For further analyses, patients with proven, probable, and possible CAPA were designated to the CAPA group. Patients were classified to the CAPA excluded group when they had >1 negative mycological test according to 2020 ECMM/ISHAM classification consensus criteria (*11*). Patients who did not undergo any of the mycological tests were designated to the CAPA not classifiable group. *Value includes 6 patients in whom CAPA was excluded at the time of autopsy. CAPA, COVID-19–associated pulmonary aspergillosis; COVID-19, coronavirus disease; ECMM/ISHAM, European Confederation for Medical Mycology/International Society for Human and Animal Mycology; ICU, intensive care unit.

We applied ECMM/ISHAM classification criteria for CAPA (*11*). We considered bronchial lavage (BL) equivalent to bronchoalveolar lavage (BAL). We assumed all CAPA classified patients demonstrated clinical factors and radiographic abnormalities. We defined 3 patient groups: CAPA, CAPA-excluded, and CAPA not classifiable (Figure 1; Appendix).

We included 519 patients in the discovery cohort; median age was 64 years, 73% were male, and 82% required invasive mechanical ventilation during ICU admission (Table 1; Appendix Table 2, 3, 4). Among patients in the discovery cohort, 279 (54%) were classifiable: 6 (2%) as CAPA proven, 32 (12%) as probable CAPA, and 4 (1%) as possible CAPA (Figure 1, panel A; Appendix Results, Tables 5, 6). CAPA incidence among classifiable patients was 15% (42/279); 85% were CAPA-excluded. Among patients in the discovery cohort, 46% (240/519) were not classifiable, including 3 who did not fulfill the criteria for possible CAPA (Figure 1, panel A). In patients with any EORTC/MSGERC host factor, CAPA incidence was 30% (13/44), compared with 16% (26/161) in patients with no host factors (p = 0.053).

**Table 1:** Demographic, clinical, and mycological characteristics of the discovery cohort in a multinational observational study of COVID-19–associated pulmonary aspergillosis in 3 countries in Europe, 2020*

**Characteristics**	Total population, n = 519	CAPA, n = 42	CAPA excluded, n = 237	p value
Age, y	64 (55–72)	68 (61–73)	65 (57–71)	0.12
Sex				
F	141 (27)	8 (19)	58 (24)	
M	378 (73)	34 (81)	179 (76)	0.56
BMI, kg/m^2^	27.2 (24.4–31.0); n = 507	27.4 (23.6–30.2); n = 40	26.9 (24.4–30.9); n = 231	0.72
Underlying conditions				
Cardiovascular disease†	291 (56)	25 (60)	130 (55)	0.62
Diabetes mellitus	139 (27)	9 (21)	61 (26)	0.70
Asthma	37 (7)	1 (2)	19 (8)	0.33
COPD	44 (9)	8 (19)	19 (8)	**0.042**
Liver cirrhosis	6 (1)	0	2 (0.8)	1.00
Rheumatological disease	31 (6)	5 (12)	14 (6)	0.18
HIV/AIDS	6 (1)	3 (7)	1 (0.4)	**0.011**
Solid organ malignancy	28 (5)	3 (7)	11 (5)	0.45
EORTC/MSGERC host factors				
Any‡	70 (16); n = 426	13 (33); n = 39	31 (19); n = 166	0.053
Recent neutropenia§	7 (2); n = 413	1 (3); n = 38	5 (3); n = 156	1.00
Hematologic malignancy	18 (4)	4 (10)	9 (4)	0.11
Receipt of allogeneic SCT	4 (0.8); n = 516	0	3 (1); n = 236	1.00
Receipt of SOT	6 (1)	1 (2)	2 (0.8)	0.39
Systemic corticosteroids <30 d before ICU admission, any dose	38 (9); n = 430	7 (18); n = 39	14 (9); n = 160	0.14
T or B cell immunosuppressants other than corticosteroids <90 d before ICU admission	31 (6); n = 514	7 (17)	12 (5); n = 233	**0.014**
Inherited severe immunodeficiency	0; n = 517	0	0; n = 236	NA
ICU treatment data				
Invasive mechanical ventilation	423 (82); n = 517	40 (98); n = 41	225 (95)	0.70
No. invasive ventilation days¶	14 (9–24); n = 395	16 (13–27); n = 37	18 (11–30); n = 212	0.98
RRT during ICU admission	93 (18); n = 516	17 (41)	44 (19); n = 236	**0.004**
Systemic corticosteroids during ICU admission	216 (42); n = 516	20 (48)	131 (56); n = 236	0.40
Outcome data				
ICU death	154 (30); n = 518	22 (52)	81 (34)	**0.036**
ICU LOS, d#	14 (8–24); n = 491	18 (12–27); n = 39	20 (12–32); n = 222	0.84
Mycologic diagnostic tests				
Serum GM OD >0.5, no. positive (%); no. values reported/no. performed	3 (2); 134/176	3 (11); 28/28	0; 106/148	NA
Serum GM OD**	0.10 (0.10–0.10); n = 134	0.10 (0.06–0.14); n = 28	0.10 (0.10–0.10); n = 106	0.95
Positive BALF/BL culture	17 (10); n = 166	17 (42); n = 41	0; n = 125	NA
BALF/BL GM OD >1.0, no. positive (%); no. OD values reported/no. BL/BALF performed	32 (19); 90/166	32 (78); 34/41	0; 55/125	NA
BALF/BL GM OD**	0.20 (0.10–1.50); n = 90	1.80 (1.00–3.90); n = 35	0.10 (0.10–0.20); n = 55	**<0.001**
Positive BALF/BL PCR, any C_t_, no. positive (%); no. reported/no. tested	9 (5); 11/166	7 (17); 7/41	2 (2); 4/125††	NA
Days between ICU admission and first positive mycologic test‡‡	NA	6 (3–9); n = 41	NA	NA

*Data are presented as no. (%) or median (IQR) unless otherwise indicated. Continuous variables were compared by Mann-Whitney U test, categorical variables by Fisher exact test with omission of missing data, unless stated otherwise. Total percentages might not equal 100% because of rounding. Bold text indicates statistical significance. BAL, bronchoalveolar lavage; BALF, BAL fluid; BL, bronchial lavage; BMI, body mass index; CAPA, COVID-19–associated pulmonary aspergillosis; COPD, chronic obstructive pulmonary disease; COVID-19, coronavirus disease; C_t_, cycle threshold; CT, computed tomography; ECMO, extracorporeal membrane oxygenation; EORTC/MSGERC, European Organization for Research and Treatment of Cancer and the Mycoses Study Group Education and Research Consortium; GM, galactomannan; ICU, intensive care unit; IQR, interquartile range; LOS, length of stay; NA, not applicable; NBL, nonbronchoscopic lavage; OD, optical density; RRT, renal replacement therapy; SCT, stem cell transplantation; SOT, solid organ transplant. †Includes hypertension ‡Includes any use of systemic corticosteroids before ICU admission; If data on one or more EORTC host factors were missing, then data were regarded as missing for this variable. §Neutropenia includes absolute neutrophil count of <0.5 × 10**^9^** cells/L for >10 d. ¶If transferred to another hospital from ICU and still on ventilatory support of any kind, duration of invasive mechanical ventilatory support was regarded as missing data and not included in the analyses. The same holds true for those who received a tracheostomy for a prolonged weaning trajectory. #Data on ICU LOS were regarded as missing if transfer to another hospital was the reason for ICU discharge because exact ICU LOS was unknown. **When multiple values were reported for 1 patient, the median of these values was used for further calculations. ††Positive PCR with C_t_ values >36 as only positive mycologic criterion. ‡‡Mycologic test considered a criterion for proven, probable, or possible CAPA according to the 2020 European Confederation for Medical Mycology/International Society for Human and Animal Mycology classification (*11*).

Chronic obstructive pulmonary disease (COPD; p = 0.04) and HIV/AIDS (p = 0.01) were more prevalent in CAPA patients (Table 1; Appendix Table 2). Among CAPA patients, 33% had >1 EORTC/MSGERC host factor, compared with 19% of CAPA-excluded patients (p = 0.053). Corticosteroid use was not more prevalent in the CAPA group (p = 0.14), in contrast to other immunosuppressant drugs (p = 0.01). In logistic regression analysis, corticosteroid use at any dose before or during ICU admission was not independently associated with CAPA development. However, COPD, HIV/AIDS, and use of other immunosuppressant drugs before ICU admission were associated with CAPA (Appendix Figure 1, panel A).

Among CAPA patients who underwent BAL or BL, *Aspergillus* culture was positive in 42%, GM was positive (optical density [OD] >1.0) in 78%, and *Aspergillus* PCR was positive in 17%. Among CAPA patients who underwent nonbronchoscopic lavage, 67% had positive cultures. Serum GM was positive in 11% of tested CAPA patients. Median time between ICU admission and first positive mycologic test was 6 (interquartile range [IQR] 3–9) days (Table 1; Appendix Table 7).

**Figure 2:**
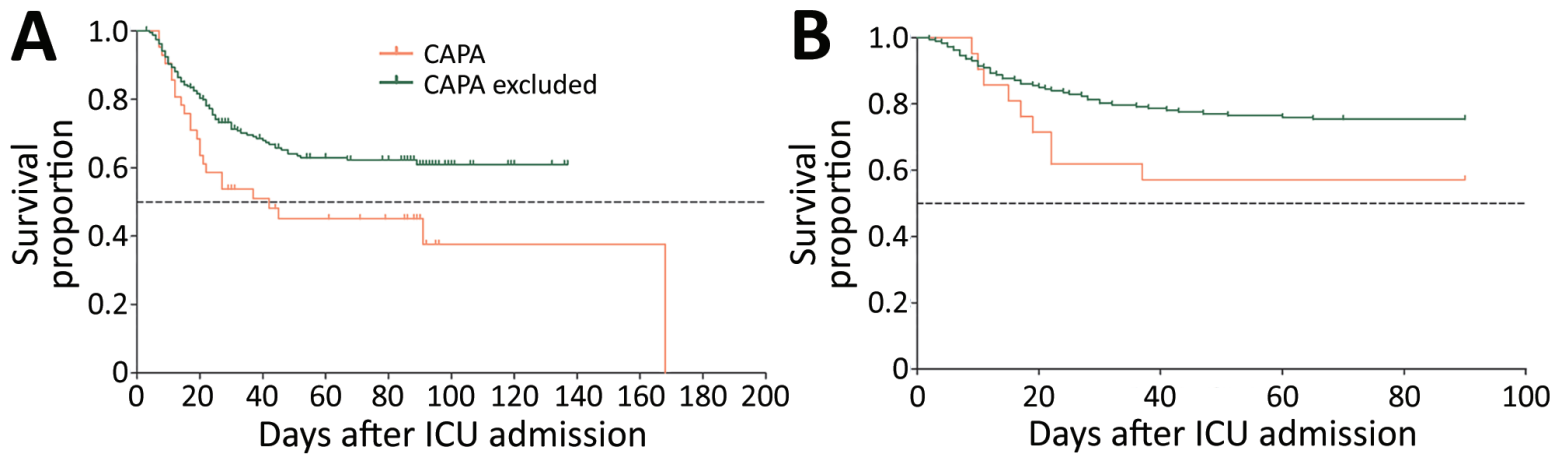
Kaplan-Meier survival curves comparing patients with CAPA and those classified as CAPA excluded in a multinational observational study. A) Discovery cohort; B) validation cohort. Survival analysis performed by using Mantel-Cox log rank test. Survival over time differs significantly in the discovery cohort (n = 279); median estimated survival in the CAPA group is 42.0 days (p = 0.015 by log rank test). In the validation cohort (n = 209), survival over time is not significantly different between the 2 groups (p = 0.065 by log rank test). CAPA, COVID-19–associated pulmonary aspergillosis; COVID-19, coronavirus disease; ICU, intensive care unit.

The proportion of patients receiving systematic corticosteroid treatment in ICUs was not significantly different between CAPA and CAPA-excluded groups (p = 0.40), nor was corticosteroid dose (p = 0.88) (Table 1; Appendix Table 4). Antifungal treatment was administered to 16% (83/519) of patients, 88% of CAPA patients, and 15% of CAPA-excluded patients (Appendix Table 8). ICU mortality rates were significantly higher in CAPA patients (52%) than in CAPA-excluded patients (34%) (p = 0.04; Table 1; Appendix Table 4); mortality rates were 67% for patients with positive serum GM. CAPA patients demonstrated reduced survival (p = 0.02) (Figure 2, panel A); estimated median survival was 42 days after ICU admission. When correcting for covariates, CAPA was not independently associated with ICU mortality rates, but older age and acute kidney injury (AKI) during ICU stay were (Appendix Figure 1, panel B).

We included 304 patients in the validation cohort (Figure 1, panel B); median age was 63 years, 25% were male, and 76% required invasive mechanical ventilation (Table 2; Appendix Tables 9, 10). Ultimately, 209/304 (69%) patients were classifiable for CAPA: 21 (10%) probable CAPA and 188 (90%) CAPA excluded (Figure 1, panel B; Appendix Results, Tables 5, 11). Among patients with EORTC/MSGERC host factors, CAPA incidence was 13% (3/23), compared with 10% (18/186) among patients without host factors (p = 0.71).

**Table 2:** Demographic, clinical, and mycological characteristics of the validation cohort in a multinational observational study of COVID-19–associated pulmonary aspergillosis in 3 countries in Europe, 2020*

Characteristics	Total population, n = 304	CAPA, n = 21	CAPA excluded, n = 188	p value
Age, y	63 (55–71)	67 (59–75)	62 (53–69)	0.06
Sex				
F	227 (75)	21 (100)	141 (75)	
M	77 (25)	0	47 (25)	**0.005**
BMI, kg/m^2^	30.0 (26.0–34.4); n = 296	30.2 (26.1–32.8); n = 20	30.0 (26.4–34.5); n = 185	0.84
Underlying conditions				
Active hematologic malignancy	10 (3)	0	6 (3)	1.00
Cardiovascular disease†	185 (61)	17 (81)	112 (60)	0.06
Diabetes mellitus	92 (30)	9 (43)	62 (33)	0.47
Asthma	22 (7)	2 (10)	12 (6)	0.64
COPD	20 (7)	2 (10)	12 (6)	0.64
Liver cirrhosis‡	5 (2)	2 (10)	2 (1)	0.051
Autoimmune disease	16 (5)	2 (10)	11 (6)	0.63
HIV/AIDS	3 (1)	0	1 (0.5)	1.00
Active solid organ malignancy	4 (1)	1 (5)	3 (2)	0.35
Bronchiectasis	5 (2)	2 (10)	1 (0.5)	**0.027**
EORTC/MSGERC host factors				
Any§	35 (12)	3 (14)	20 (11)	0.71
Recent neutropenia¶	0; n = 303	0	0; n = 187	NA
Hematological malignancy	10 (3)	0	6 (3)	1.00
Receipt of SOT	9 (3)	1 (5)	5 (3)	0.48
Corticosteroids >0.3 mg/kg for >3 wks within previous 60 d	17 (6)	2 (10)	10 (5)	0.34
Other immunosuppressants <90 d before ICU admission	23 (8)	2 (10)	16 (9)	0.70
ICU treatment data				
Invasive mechanical ventilation	228 (76); n = 302	19 (95); n = 20	168 (89)	0.70
No. invasive ventilation days	15 (9–25); n = 212	18 (13–25); n = 17	15 (9–25); n = 157	0.21
RRT	64 (21); n = 303	11 (55); n = 20	47 (25)	**0.008**
Systemic corticosteroids during ICU admission	147 (49); n = 303	11 (52)	106 (57); n = 187	0.82
Outcome data				
ICU death	69 (23); n = 299	9 (43)	46 (25); n = 185	0.12
ICU LOS, d#	14 (8–26); n = 295	22 (12–35); n = 20	18 (10–28); n = 183	0.27
Mycologic diagnostic tests				
Serum GM OD >0.5	4 (2); n = 172**	4 (22); n = 18	0; n = 154††	NA
Serum GM OD	0.07 (0.04–0.12); n = 172**	0.10 (0.06–0.34); n = 18	0.06 (0.04–0.11); n = 154††	**0.008**
Positive BALF culture	11 (8); n = 135	11 (52) n = 21	0; n = 114	NA
BALF GM OD >1.0	13 (11); n = 123	13 (62) n = 21	0; n = 102	NA
BALF GM OD‡‡	0.12 (0.05–0.32); n = 123	1.10 (0.12–3.06); n = 21	0.11 (0.05–0.18); n = 102	**<0.001**
Positive BALF PCR, any C_t_	8 (13); n = 64	8 (53); n = 15	0; n = 49	NA
Serum β-D-glucan value **≥**80 pg/mL	37 (20); n = 184	8 (42); n = 19	29 (18); n = 160	**0.030**
Serum β-D-glucan value§§	31 (13–60); n = 184	34 (31–156); n = 19	31 (10–59); n = 160	0.055

*Data are presented as no. (%) or median (IQR), unless stated otherwise. Continuous variables were compared by Mann-Whitney U test, categorical variables by Fisher exact test with omission of missing data, unless stated otherwise. Total percentages might not equal 100% because of rounding. Bold text indicates statistical significance. BAL, bronchoalveolar lavage; BALF, BAL fluid; BL, bronchial lavage; BMI, body mass index; CAPA, COVID-19–associated pulmonary aspergillosis; COPD, chronic obstructive pulmonary disease; COVID-19, coronavirus disease; Ct, cycle threshold; CT, computed tomography; ECMO, extracorporeal membrane oxygenation; EORTC/MSGERC, European Organization for Research and Treatment of Cancer and the Mycoses Study Group Education and Research Consortium; ICU, intensive care unit; IQR, interquartile range; GM, galactomannan; LOS, length of stay; NA, not applicable; OD, optical density; RRT, renal replacement therapy; SAPS, simplified acute physiology score; SOT, solid organ transplant; TBA, tracheobronchial aspirate. †Includes Factor V Leiden mutation and hypertension. ‡Includes hemochromatosis. §Includes use of any systemic corticosteroids. We did not assess receipt of an allogeneic stem cell transplant, presence of an inherited severe immunodeficiency, and presence of acute graft-versus-host disease. ¶Neutropenia includes absolute neutrophil count of <0.5 × 10**^9^**/L for >10 d. #Data on ICU LOS were regarded as missing if still admitted at the time of data entry or if transfer to another hospital was the reason for ICU discharge. **Serum GM performed in 173 patients, including 1 patient with an unknown result. ††Serum GM values known for 154 patients, unknown value in 1 patient. ‡‡One value of >6.0 entered as 6.0. §§One value of >500 pg/mL entered as 500 pg/mL.

All 21 probable CAPA patients were female; cardiovascular disease, excluding hypertension (p = 0.02), and bronchiectasis (p = 0.03) were more prevalent in this group (Table 2; Appendix Table 9). Use of corticosteroids before or during ICU admission or other immunosuppressant drugs before ICU admission were not independently associated with CAPA (Appendix Figure 1, panel C). In the validation cohort, 19% received antifungal treatment; 57% of the CAPA group received antifungal treatment (Appendix Table 8).

Corticosteroid use during ICU stay was not significantly different between the CAPA and CAPA-excluded groups (p = 0.82) in the validation cohort. ICU mortality rates were higher in the CAPA group than the CAPA-excluded group (43% vs. 25%; p = 0.12) (Table 2; Figure 2, panel B; Appendix Table 10). The ICU mortality rate was 50% in patients with positive serum GM. CAPA was not independently associated with ICU death, but older age and AKI during ICU admission were (Appendix Table 10, Figure 1, panel D).

## Conclusions

We found CAPA incidence was 10%–15%, corresponding to the 14%–19% reported in other studies (*8*,*9*). Discovery cohort CAPA incidence was similar to influenza-associated pulmonary aspergillosis (IAPA) incidence in ICUs (*12*,*13*). CAPA seems to develop later after ICU admission than IAPA. Median time to first positive mycologic test in our study was 6 days after ICU admission, similar to other studies reporting 4–8 days (*7*–*9*) but in contrast to the median 3 days reported for IAPA (*12*,*14*).

Corticosteroids were not associated with CAPA in our study, consistent with previous reports (*7*–*9*), but contrasting associations seen with invasive pulmonary aspergillosis (IPA) and IAPA (*12*). This finding might be explained by possible dual effects of corticosteroids in COVID-19, impairing anti-*Aspergillus* immunity while simultaneously ameliorating the hyperinflammatory immune dysregulation and associated tissue damage conducive to IPA.

We found CAPA ICU mortality rates were 43%–52%, in line with previous reports (*7*–*9*) and comparable to those for IAPA (*12*). We could not assess antifungal treatment effects on mortality rates, but CAPA patients in the validation cohort who received antifungal treatment demonstrated a trend toward improved survival (Appendix Figure 2).

The first limitation of our study is that assuming clinical and imaging factors were available for all patients classified with CAPA possibly led to overreporting of CAPA. Excluding CAPA based on 1 negative mycologic test might have led to underreporting. Another limitation was that patients undergoing mycologic workup were likely more severely ill, which becomes apparent when comparing baseline and outcome data of the CAPA not classifiable group to the other 2 groups (Appendix Tables 5–12). Several classifications have been published or updated after we initiated this study; therefore, not all diagnostic modalities were evaluated, and we used some terms, such as BAL and BL, interchangeably (*11*,*15*).

In conclusion, we report CAPA incidence of 10%–15% in COVID-19 patients admitted to ICUs, CAPA ICU mortality rates of 43%–52%, and decreased survival over time. Clinicians should be aware of CAPA and that underlying factors, including COPD, immunosuppressant drugs other than corticosteroids, and HIV/AIDS, can increase the risk for CAPA.

Appendix Additional information on a multinational cohort study of COVID-19–associated pulmonary aspergillosis.

## References

[1] Koehler P, Cornely OA, Böttiger BW, Dusse F, Eichenauer DA, Fuchs F, et al. COVID-19 associated pulmonary aspergillosis. Mycoses. 2020;63:528–34. 10.1111/myc.13096 32339350 PMC7267243

[2] van Arkel ALE, Rijpstra TA, Belderbos HNA, van Wijngaarden P, Verweij PE, Bentvelsen RG. COVID-19–associated pulmonary aspergillosis. Am J Respir Crit Care Med. 2020;202:132–5. 10.1164/rccm.202004-1038LE 32396381 PMC7328331

[3] Rutsaert L, Steinfort N, Van Hunsel T, Bomans P, Naesens R, Mertes H, et al. COVID-19-associated invasive pulmonary aspergillosis. Ann Intensive Care. 2020;10:71. 10.1186/s13613-020-00686-4 32488446 PMC7265874

[4] Wang J, Yang Q, Zhang P, Sheng J, Zhou J, Qu T. Clinical characteristics of invasive pulmonary aspergillosis in patients with COVID-19 in Zhejiang, China: a retrospective case series. Crit Care. 2020;24:299. 10.1186/s13054-020-03046-7 32503617 PMC7274513

[5] Alanio A, Dellière S, Fodil S, Bretagne S, Mégarbane B. Prevalence of putative invasive pulmonary aspergillosis in critically ill patients with COVID-19. Lancet Respir Med. 2020;8:e48–9. 10.1016/S2213-2600(20)30237-X 32445626 PMC7239617

[6] Lamoth F, Glampedakis E, Boillat-Blanco N, Oddo M, Pagani JL. Incidence of invasive pulmonary aspergillosis among critically ill COVID-19 patients. Clin Microbiol Infect. 2020;26:1706–8. 10.1016/j.cmi.2020.07.010 32659385 PMC7348600

[7] Bartoletti M, Pascale R, Cricca M, Rinaldi M, Maccaro A, Bussini L, et al.; PREDICO study group. Epidemiology of invasive pulmonary aspergillosis among COVID-19 intubated patients: a prospective study. Clin Infect Dis. 2020;ciaa1065; Epub ahead of print. 10.1093/cid/ciaa1065 32719848 PMC7454393

[8] White PL, Dhillon R, Cordey A, Hughes H, Faggian F, Soni S, et al. A national strategy to diagnose COVID-19–associated invasive fungal disease in the intensive care unit. [Epub ahead of print]. Clin Infect Dis. 2020. 10.1093/cid/ciaa1298 PMC7499527 32860682

[9] Dellière S, Dudoignon E, Fodil S, Voicu S, Collet M, Oillic PA, et al. Risk factors associated with COVID-19-associated pulmonary aspergillosis in ICU patients: a French multicentric retrospective cohort. Clin Microbiol Infect. 2020;27:790.e1–5. 10.1016/j.cmi.2020.12.005 33316401 PMC7733556

[10] Verweij PE, Gangneux JP, Bassetti M, Brüggemann RJM, Cornely OA, Koehler P, et al.; European Confederation of Medical Mycology; International Society for Human and Animal Mycology; European Society for Clinical Microbiology and Infectious Diseases Fungal Infection Study Group; ESCMID Study Group for Infections in Critically Ill Patients. Diagnosing COVID-19-associated pulmonary aspergillosis. Lancet Microbe. 2020;1:e53–5. 10.1016/S2666-5247(20)30027-6 32835328 PMC7211496

[11] Koehler P, Bassetti M, Chakrabarti A, Chen SCA, Colombo AL, Hoenigl M, et al.; European Confederation of Medical Mycology; International Society for Human Animal Mycology; Asia Fungal Working Group; INFOCUS LATAM/ISHAM Working Group; ISHAM Pan Africa Mycology Working Group; European Society for Clinical Microbiology; Infectious Diseases Fungal Infection Study Group; ESCMID Study Group for Infections in Critically Ill Patients; Interregional Association of Clinical Microbiology and Antimicrobial Chemotherapy; Medical Mycology Society of Nigeria; Medical Mycology Society of China Medicine Education Association; Infectious Diseases Working Party of the German Society for Haematology and Medical Oncology; Association of Medical Microbiology; Infectious Disease Canada. Defining and managing COVID-19-associated pulmonary aspergillosis: the 2020 ECMM/ISHAM consensus criteria for research and clinical guidance. Lancet Infect Dis. 2021;21:e149–62. 10.1016/S1473-3099(20)30847-1 33333012 PMC7833078

[12] Schauwvlieghe AFAD, Rijnders BJA, Philips N, Verwijs R, Vanderbeke L, Van Tienen C, et al.; Dutch-Belgian Mycosis study group. Invasive aspergillosis in patients admitted to the intensive care unit with severe influenza: a retrospective cohort study. Lancet Respir Med. 2018;6:782–92. 10.1016/S2213-2600(18)30274-1 30076119

[13] van de Veerdonk FL, Kolwijck E, Lestrade PP, Hodiamont CJ, Rijnders BJ, van Paassen J, et al.; Dutch Mycoses Study Group. Influenza-associated aspergillosis in critically ill patients. Am J Respir Crit Care Med. 2017;196:524–7. 10.1164/rccm.201612-2540LE 28387526

[14] Wauters J, Baar I, Meersseman P, Meersseman W, Dams K, De Paep R, et al. Invasive pulmonary aspergillosis is a frequent complication of critically ill H1N1 patients: a retrospective study. Intensive Care Med. 2012;38:1761–8. 10.1007/s00134-012-2673-2 22895826 PMC7079899

[15] Donnelly JP, Chen SC, Kauffman CA, Steinbach WJ, Baddley JW, Verweij PE, et al.; Revision and Update of the Consensus Definitions of Invasive Fungal Disease from the European Organization for Research and Treatment of Cancer and the Mycoses Study Group Education and Research Consortium. Revision and Update of the Consensus Definitions of Invasive Fungal Disease From the European Organization for Research and Treatment of Cancer and the Mycoses Study Group Education and Research Consortium. Clin Infect Dis. 2020;71:1367–76. 10.1093/cid/ciz1008 31802125 PMC7486838

