# Supplementary material for: Multinational Observational Cohort Study of COVID-19–Associated Pulmonary Aspergillosis
Source: Emerg Infect Dis. 2021 Nov;27(11):2892–8. doi: 10.3201/eid2711.211174 (PMC8544971; doi:10.3201/eid2711.211174)
Supplement: Appendix — Additional information on a multinational cohort study of COVID-19–associated pulmonary aspergillosis. [file 21-1174-Techapp-s1.pdf]

Article DOI: https://doi.org/10.3201/eid2711.211174

Multinational Observational Cohort Study of COVID-19–Associated Pulmonary Aspergillosis

Appendix

Additional Methods

Study Design and Participants

Data on the discovery cohort were largely collected prospectively and partially retrospectively; patients were admitted during February 28–May 27, 2020. Data on the validation cohort were collected retrospectively; patients in this cohort were admitted during April 7–May 31, 2020. The 2 cohorts were analyzed separately because of differences in inclusion criteria and data variables collected. The number of patients included per center varied (Appendix Table 1). Patients were followed for 90 days after ICU admission or until ICU discharge, whichever occurred last.

Ethics Statement

This study was performed in accordance with the latest version of the Declaration of Helsinki (https://www.wma.net), the International Conference on Harmonisation–Good Clinical Practice (ICH-GCP) guidelines (https://ichgcp.net) and local legislation and regulations. For all centers in the Netherlands, ethical approval was granted by the Ethics Board region Arnhem-Nijmegen; based on the observational nature of this study, informed consent was waived (approval no. CMO 2020–6339).

For centers in Belgium, ethical approval was granted by the University Hospitals Leuven Ethics Board (approval no. S64071) and local ethics boards of participating centers. Ethical approval for the separate study protocol for the sites in France was granted by the institutional committee of the Amiens University Hospital (registration no. PI2020_843_0028.). The study protocol was registered with the Commission Nationale de l’Informatique et des Libertés, France.

Data Collection

For the discovery cohort, local investigators collected pseudonymized patient data from medical files into Castor Electronic Data Capture (Castor EDC, https://www.castoredc.com) electronic case report form (eCRF). Pseudonymized data for the validation cohort were entered into a SharePoint eCRF (Department of Clinical Research, Amiens University Hospital, Amiens, France).

Definitions

Clinically presumed coronavirus disease 2019 (COVID-19) was based on clinical signs and symptoms and exposure to severe acute respiratory syndrome coronavirus 2 (SARS-CoV-2), as assessed by treating physicians or multidisciplinary COVID-19 team. COVID-19–associated pulmonary aspergillosis (CAPA) diagnosis was based on the 2020 European Confederation for Medical Mycology/International Society for Human and Animal Mycology (ECMM/ISHAM) consensus classification (*1*). We applied several necessary modifications needed because of the eCRF's design and differences in interpretation between participating centers regarding performance of bronchial lavage (BL) or bronchoalveolar lavage (BAL). Although a BL was intended to signify a nonbronchoscopic, nondirected bronchial lavage (NBL) and a BAL a directed, bronchoscopic lavage, these terms were used differently by several centers. To avoid missing any directed BALs performed, we regarded BLs as equivalent to BALs during data analysis. Due to missing data on clinical factors and radiological results in many patients, all patients classified with CAPA were considered to demonstrate these clinical characteristics and (infiltrative) abnormalities on thoracic imaging during ICU stay. Patients who underwent none of the mycological tests required for classification as proven or probable CAPA, including autopsy, were designated CAPA not classifiable. Patients who underwent mycological tests were further evaluated for the presence of CAPA. In the validation cohort, data were not available to classify patients as possible CAPA.

According to the 2020 ECMM/ISHAM classification (*1*), BL or BALF galactomannan (GM) results that were qualitatively positive without a known quantitative result were not regarded as a positive mycological result. Patients were classified into 3 defined groups for further analyses. The CAPA group comprised patients with proven, probable, or possible *Aspergillus* tracheobronchitis, pulmonary CAPA, or both. The CAPA excluded group comprised patients who underwent diagnostic workup, but had no evidence for proven, probable, or

possible CAPA, including no CAPA at autopsy, or patients without probable CAPA, but who were not classifiable as possible CAPA. The CAPA not classifiable group included patients who did not undergo any required mycological testing for proven or probable CAPA or those who were not classifiable for probable CAPA but had possible CAPA excluded.

We defined acute kidney injury (AKI) according to the Kidney Disease: Improving Global Outcomes (KDIGO; https://kdigo.org) criteria. AKI criteria include increase in serum creatinine of ≥26.5 µmol/L (≥0.3 mg/dL) within 48 hours or increase in serum creatinine ≥1.5 times baseline values, which is known or presumed to have occurred within the prior 7 days or urine volume <0.5 mL/kg/h for 6 hours (*2*). Prior to classification, correction of volume status and obstructive causes of AKI are allowed.

We also defined stages of AKI according to the KDIGO criteria. Patients were classified according to the criteria that resulted in the highest, that is the most severe, stage of injury. Stage 1 is increase in serum creatinine to 1.5–1.9 times baseline values or increase in serum creatinine by ≥26.5 µmol/L (≥0.3 mg/dL) or reduction in urine output to <0.5 mL/kg/hour for 6–12 hours. Stage 2 is increase in serum creatinine to 2.0–2.9 times baseline values, or reduction in urine output to <0.5 mL/kg/hour for ≥12 hours. Stage 3 is increase in serum creatinine to 3.0 times baseline values, or increase in serum creatinine to ≥353.6 µmol/L (≥4.0 mg/dL), or reduction in urine output to <0.3 mL/kg/h for ≥24 hours, or anuria for ≥12 hours, or the initiation of renal replacement therapy.

Comparisons between CAPA Classification Criteria

CAPA diagnosis according to the 2020 ECMM/ISHAM classification was compared to the IAPA expert opinion case definition (*3*), the modified *Asp*ICU (m*Asp*ICU) classification (*4*,*5*), and the revised European Organization for Research and Treatment of Cancer and the Mycoses Study Group Education and Research Consortium (EORTC/MSGERC) classification (*6*). As we did for the ECMM/ISHAM classification, we presumed all patients classified with IPA had clinical factors, signs, symptoms, and pulmonary infiltrates on thoracic imaging present for all other classifications, if applicable. As such, only subset A of the IAPA expert opinion case definition criteria for probable IPA could be evaluated in our cohorts. For the m*Asp*ICU classification, BAL fluid (BALF) GM optical density (OD) ≥1.0 and serum GM OD ≥0.5 were added as mycological criteria for the diagnosis of putative IPA and positive *Aspergillus* BALF

culture was regarded as an entry criterion. For EORTC/MSGERC definitions, the eCRF was not designed to assess the presence of acute graft-versus-host-disease; therefore, we could not take this host factor into account. Due to frequent missing data regarding details on use of corticosteroids before admission to the ICU, all systemic corticosteroid use was considered a risk factor for invasive aspergillosis according to m*Asp*ICU and EORTC/MSGERC criteria. We compared the 2020 ECMM/ISHAM CAPA classification with the clinically reported occurrence of CAPA in patients' medical files, that is physician reported CAPA, regardless of fulfilment of any formal classification criteria.

Statistical Analysis

All data are expressed as no. (%) or median (interquartile range [IQR]). We compared variables by using Fisher exact test, Mann-Whitney U test, or Kruskal-Wallis test, as appropriate. We analyzed survival differences by using the Kaplan-Meier method and log rank test. We performed binary logistic regression analysis to detect independent predictors of CAPA occurrence and ICU death. In all cases, we considered $p<0.05$ statistically significant. We did not apply corrections for multiple statistical testing during these analyses; readers should keep this in mind when interpreting results.

For binary logistic regression analysis, we used different independent variables as potential predictors for CAPA occurrence and ICU death. For CAPA occurrence, we used underlying conditions significantly more prevalent in the CAPA group in univariate analysis and corticosteroid use before and during ICU admission as independent variables. For ICU death, age, sex, AKI, renal replacement therapy (RRT), mechanical ventilation, use of vasopressors and/or inotropes and corticosteroids during ICU admission and presence of CAPA were used as independent variables or covariates. We performed statistical analyses by using SPSS Statistics for Windows version 25.0 (IBM Corp., https://www.ibm.com) or GraphPad Prism 5.03 for Windows (GraphPad Software Inc., https://www.graphpad.com).

Results

Discovery Cohort

Of the 521 patients admitted to the participating ICUs in the Netherlands and Belgium during the study period, 1 patient was excluded from further analysis because <60% of data were

collected due to an ICU stay of only several hours, at which point a non-ICU policy was instated and patient was discharged from the ICU. Another patient was excluded because of objection to participation (Figure 1, panel A).

Of the 519 included patients, 4 (0.8%) did not have a positive SARS-CoV-2 PCR result, 2 of whom later were shown to be SARS-CoV-2 IgG positive. Three of the SARS-CoV-2 PCR–negative patients had radiographic findings on thoracic computed tomography (CT) and 1 on thoracic x-ray suggestive of COVID-19. One patient (0.2%) had no known SARS-CoV-2 PCR results but had radiographic findings on thoracic CT suggestive of COVID-19.

ECMM/ISHAM CAPA Classification

In the discovery cohort, tracheobronchitis could be evaluated in 187 patients who underwent bronchoscopy with or without BAL or BL, underwent autopsy, or both. Diagnostic tests to classify patients as proven tracheobronchitis or pulmonary CAPA were performed in 41/519 (8%) patients. Tests for classification as probable (pulmonary) CAPA were performed in 273/519 (53%) patients, and tests for classification as possible CAPA in 43/519 (8%). Because positive culture, GM and PCR results were reported more readily than negative ones, we could not give exact total numbers of fungal culture, GM testing, and *Aspergillus* PCR performed in BAL, BL, and NBL samples obtained (Tables 1, 2; Appendix Tables 7, 12).

In the validation cohort, 127/304 (42%) of patients could be evaluated for invasive *Aspergillus* tracheobronchitis, and 209/304 (69%) could be evaluated for probable CAPA. Data to classify patients as possible CAPA were not collected in this cohort.

CAPA Patients with COPD or Bronchiectasis

The discovery cohort contained 8 patients with CAPA and underlying COPD, whereas the validation cohort contained 2 CAPA patients with COPD. Furthermore, the validation cohort included 2 patients with CAPA and bronchiectasis. Data on bronchiectasis were not collected in the discovery cohort. The 2020 ECMM/ISHAM classification requires a positive GM test result as confirmation of a positive *Aspergillus* culture or PCR result in patients with COPD or another chronic respiratory disease to rule out colonization or chronic aspergillosis (*1*). Of the 12 patients with COPD or bronchiectasis in both cohorts, 11 (92%) had a BAL or BL GM OD $\geq$1.0, and 1 had a GM OD >6.0 in an NBL sample, indicating high fungal load with hyphal formation in the respiratory tract and probably not mere colonization.

Discovery Cohort Microbiological Results

Among the 17 patients with positive BL or BAL cultures, *A. fumigatus* was found in 15/17 (88%), *A. nidulans* in 1 (6%) patient, and both *A. fumigatus* and *A. flavus* in 1 (6%) patient. None of the 5 patients for whom susceptibility data were available demonstrated voriconazole resistance. Among 9 patients in whom positive BAL or BL PCR results were reported, *A. fumigatus* was reported in 3 (33%); speciation was not possible or provided in the other 6 (67%). Azole resistance PCR test results were reported in 1 patient in whom wild-type *A. fumigatus* was found. *A. fumigatus* was found in all 6 patients with positive NBL cultures; 1 patient had a positive BALF culture and a positive NBL culture on different dates and both demonstrated *A. fumigatus*. In 5 of these patients, an *Aspergillus* PCR also was performed on the same NBL sample: in 3 patients *A. fumigatus* was found and no species were identified in the other 2.

Logistic Regression Analysis

In the discovery cohort, RRT was more prevalent in the CAPA group than in the CAPA excluded group. To account for any possible effects of this difference on ICU death, we explored an interaction term between RRT and CAPA in the logistic regression model, which demonstrated no interaction between the 2 variables (adjusted odds ratio [aOR] for the interaction term for ICU death 1.42, 95% CI 0.30–6.87, $p = 0.66$). In the validation cohort, we explored an interaction term for AKI during ICU admission and CAPA for the occurrence of ICU death, which also demonstrated no interaction between the 2 variables for ICU death (aOR 0.16, 95% CI 0.01–2.71, $p = 0.20$).

References

1. Koehler P, Bassetti M, Chakrabarti A, Chen SCA, Colombo AL, Hoenigl M, et al.; European Confederation of Medical Mycology; International Society for Human Animal Mycology; Asia Fungal Working Group; INFOCUS LATAM/ISHAM Working Group; ISHAM Pan Africa Mycology Working Group; European Society for Clinical Microbiology; Infectious Diseases Fungal Infection Study Group; ESCMID Study Group for Infections in Critically Ill Patients; Interregional Association of Clinical Microbiology and Antimicrobial Chemotherapy; Medical Mycology Society of Nigeria; Medical Mycology Society of China Medicine Education Association; Infectious Diseases Working Party of the German Society for Haematology and Medical Oncology; Association of Medical Microbiology; Infectious Disease Canada. Defining and managing COVID-19–associated pulmonary aspergillosis: the 2020 ECMM/ISHAM consensus criteria for research and clinical guidance. Lancet Infect Dis. 2021;21;e149–62. PubMed https://doi.org/10.1016/S1473-3099(20)30847-1

2. Kellum JA, Lameire N; KDIGO AKI Guideline Work Group. Diagnosis, evaluation, and management of acute kidney injury: a KDIGO summary (Part 1). Crit Care. 2013;17:204. PubMed https://doi.org/10.1186/cc11454

3. Verweij PE, Rijnders BJA, Brüggemann RJM, Azoulay E, Bassetti M, Blot S, et al. Review of influenza-associated pulmonary aspergillosis in ICU patients and proposal for a case definition: an expert opinion. Intensive Care Med. 2020;46:1524–35. PubMed https://doi.org/10.1007/s00134-020-06091-6

4. Blot SI, Taccone FS, Van den Abeele AM, Bulpa P, Meersseman W, Brusselaers N, et al.; AspICU Study Investigators. A clinical algorithm to diagnose invasive pulmonary aspergillosis in critically ill patients. Am J Respir Crit Care Med. 2012;186:56–64. PubMed https://doi.org/10.1164/rccm.201111-1978OC

5. Schauwvlieghe AFAD, Rijnders BJA, Philips N, Verwijs R, Vanderbeke L, Van Tienen C, et al.; Dutch-Belgian Mycosis study group. Invasive aspergillosis in patients admitted to the intensive care unit with severe influenza: a retrospective cohort study. Lancet Respir Med. 2018;6:782–92. PubMed https://doi.org/10.1016/S2213-2600(18)30274-1

6. Donnelly JP, Chen SC, Kauffman CA, Steinbach WJ, Baddley JW, Verweij PE, et al.; Revision and Update of the Consensus Definitions of Invasive Fungal Disease from the European Organization for Research and Treatment of Cancer and the Mycoses Study Group Education and Research Consortium. Revision and update of the consensus definitions of invasive fungal disease from the

European Organization for Research and Treatment of Cancer and the Mycoses Study Group Education and Research Consortium. Clin Infect Dis. 2020;71:1367–76. PubMed https://doi.org/10.1093/cid/ciz1008

Appendix Table 1. Number of included patients by participating center in a multinational observational study of COVID-19–associated pulmonary aspergillosis*

Participating center	No. patients
Discovery cohort	
Amsterdam University Medical Centers, Academisch Medisch Centrum (AMC), Amsterdam, the Netherlands	80
Amsterdam University Medical Centers, Vrije Universiteit Medisch Centrum (VUmc), Amsterdam	69
Erasmus Medical Center, Rotterdam, the Netherlands	85
Radboud University Medical Center, Nijmegen, the Netherlands	80
Ziekenhuis Netwerk Antwerpen (ZNA) Campus Stuivenberg, Antwerpen, Belgium	41
Algemeen Ziekenhuis (AZ) St-Jan Brugge-Oostende, Brugge, Belgium	14
University Hospitals Leuven, Leuven, Belgium	92
Algemeen Ziekenhuis (AZ) Delta Hospital, Roeselare, Belgium	58
Validation cohort	
Amiens University Hospital, Amiens, France	119
Lille University Hospital, Lille, France	128
Rouen University Hospital, Rouen, France	57

*COVID-19, coronavirus disease.

Appendix Table 2. Demographic and clinical characteristics of the discovery cohort in a multinational observational study of COVID-19–associated pulmonary aspergillosis*

Characteristics	Total population, n = 519	CAPA, n = 42	CAPA excluded, n = 237	CAPA not classifiable, n = 240	p value†	p value‡
Age, y	64 (55–72)	68 (61–73)	65 (57–71)	64 (53–73)	0.12	0.36
Sex						
F	141 (27)	8 (19)	58 (24)	75 (31)		
M	378 (73)	34 (81)	179 (76)	165 (69)	0.56	0.13
Ethnicity	N = 365	N = 37	N = 143	N = 185		
Caucasian	294 (81)	32 (87)	115 (80)	147 (80)	0.48	0.64
Northern African	20 (6)	0 (0)	10 (7)	10 (5)	0.22	0.28
Middle Eastern	6 (2)	0 (0)	3 (2)	3 (2)	1.00	1.00
Black or sub-Saharan African	18 (5)	1 (3)	5 (4)	12 (7)	1.00	0.49
Asian	18 (5)	2 (5)	6 (4)	10 (5)	0.67	0.83
Hispanic or Latino	6 (2)	2 (5)	1 (0.7)	3 (2)	0.11	0.15
Pacific Islander	3 (0.8)	0 (0)	3 (2)	0 (0)	1.00	0.13
Unknown	154 (30); n = 519	5 (12); n = 42	94 (40); n = 237	55 (23); n = 240	**0.0004**	**<0.0001**
Height, m	1.75 (1.68–1.80); n = 507	1.77 (1.69–1.81); n = 40	1.75 (1.70–1.80); n = 231	1.74 (1.65–1.80); n = 236	0.875	0.059
Weight, kg	84.3 (74.2–95.0); n = 515	83.3 (70.0–94.3); n = 42	84.1 (75.0–95.0); n = 234	85.0 (73.8–95.0); n = 239	0.321	0.58
BMI, kg/m^2	27.2 (24.4–31.0); n = 507	27.4 (23.6–30.2); n = 40	26.9 (24.4–30.9); n = 231	27.5 (24.3–31.5); n = 236	0.72	0.64
BMI >30 kg/m^2	158 (31); n = 507	10 (25); n = 40	69 (30); n = 231	79 (34); n = 236	0.58	0.51
Smoking status	N = 308	N = 23	N = 138	N = 147		
Yes, current smoker§	25 (8)	4 (17)	9 (7)	12 (8)	0.094	0.20
No, never	140 (46)	9 (39)	53 (38)	78 (53)	1.00	**0.036**
No, but former smoker	143 (46)	10 (44)	76 (55)	57 (39)	0.369	**0.021**
Unknown	211 (41); n = 519	19 (45); n = 42	99 (42); n = 237	93 (39); n = 240	0.736	0.67
Admission and disease course data						
Days between first signs/symptoms and hospital admission	7 (5–10); n = 467	7 (4–10); n = 36	7 (5–10); n = 221	7 (5–10); n = 210	0.72	0.40
Days between hospital admission and ICU admission	1 (0–3); n = 518	2 (0–5); n = 42	2 (0–4); n = 236	1 (0–3); n = 240	0.60	0.053

Characteristics	Total population, n = 519	CAPA, n = 42	CAPA excluded, n = 237	CAPA not classifiable, n = 240	p value†	p value‡
Days between first signs/symptoms and first positive SARS-CoV-2 PCR	7 (4–10); n = 459	9 (3–10); n = 37	7 (5–11); n = 213	7 (4–10); n = 209	0.48	0.13
Reason for ICU admission						
Respiratory insufficiency	507 (98)	42 (100)	234 (99)	231 (96)	1.00	0.16
Hemodynamic instability	12 (2)	1 (2)	4 (2)	7 (3)	0.56	0.60
Decreased consciousness	12 (2)	0 (0)	5 (2)	7 (3)	1.00	0.74
Other	11 (2)	1 (2)	3 (1)	7 (3)	0.48	0.44
Underlying conditions						
Any	447 (86)	39 (93)	201 (85)	207 (86)	0.23	0.41
Acute leukemia <90 d before ICU admission	1 (0.2)	0 (0)	1 (0.4)	0 (0)	1.00	0.54
Acute leukemia >90 d before ICU admission	3 (0.6)	1 (2)	2 (0.8)	0 (0)	0.39	0.07
SCT <90 d before ICU admission	0 (0)	0 (0)	0 (0)	0 (0)	NA	NA
SCT >90 d before ICU admission	6 (1)	0 (0)	5 (2)	1 (0.4)	1.00	0.27
Other hematological malignancy	20 (4)	3 (7)	11 (5)	6 (3)	0.45	0.18
Kidney transplantation	3 (0.6)	0 (0)	1 (0.4)	2 (0.8)	1.00	1.00
Lung transplantation	1 (0.2)	1 (2)	0 (0)	0 (0)	0.15	0.08
Heart transplantation	2 (0.4)	0 (0)	1 (0.4)	1 (0.4)	1.00	1.00
Liver or pancreas transplantation	0 (0)	0 (0)	0 (0)	0 (0)	NA	NA
Cardiovascular disease¶	291 (56)	25 (60)	130 (55)	136 (57)	0.62	0.83
Diabetes mellitus	139 (27)	9 (21)	61 (26)	69 (29)	0.70	0.58
Asthma	37 (7)	1 (2)	19 (8)	17 (7)	0.33	0.50
COPD	44 (9)	8 (19)	19 (8)	17 (7)	**0.042**	0.052
Cystic fibrosis	0 (0)	0 (0)	0 (0)	0 (0)	NA	NA
Pulmonary TB	0 (0)	0 (0)	0 (0)	0 (0)	NA	NA
Multiple sclerosis	4 (0.8)	0 (0)	2 (0.8)	2 (0.8)	1.00	1.00
Liver cirrhosis	6 (1)	0 (0)	2 (0.8)	4 (2)	1.00	0.81
Ulcerative colitis	3 (0.6)	0 (0)	0 (0)	3 (1)	NA	0.31
Crohn's disease	0 (0)	0 (0)	0 (0)	0 (0)	NA	NA
Rheumatological disease	31 (6)	5 (12)	14 (6)	12 (5)	0.18	0.22
Psoriasis	7 (1)	1 (2)	3 (1)	3 (1)	0.481	0.70
HIV/AIDS	6 (1)	3 (7)	1 (0.4)	2 (0.8)	**0.011**	**0.008**
Congenital immunodeficiency syndrome	0 (0)	0 (0)	0 (0)	0 (0)	NA	NA
Acquired immunodeficiency syndrome other than HIV/AIDS	3 (0.6)	1 (2)	2 (0.8)	0 (0)	0.388	0.07
Solid organ malignancy	28 (5)	3 (7)	11 (5)	14 (6)	0.45	0.64
Other malignancy	11 (2)	0 (0)	5 (2)	6 (3)	1.00	0.90
CKD requiring RRT	7 (1)	0 (0)	0 (0)	7 (3)	NA	**0.023**
CKD not requiring RRT	30 (6)	3 (7)	8 (3)	19 (8)	0.22	0.08
Thyroid disease	14 (3)	3 (7)	8 (3)	3 (1)	0.22	0.05
Other	215 (41)	13 (31)	110 (46)	92 (38)	0.066	0.07
None	72 (14)	3 (7)	36 (15)	33 (14)	0.23	0.41
EORTC/MSGERC host factors						
Any#	70 (16); n = 426	13 (33); n = 39	31 (19); n = 166	26 (12); n = 221	0.053	**0.003**
Recent neutropenia**	7 (2); n = 413	1 (3); n = 38	5 (3); n = 156	1 (0.5); n = 219	1.00	0.09
Hematological malignancy	18 (4)	4 (10)	9 (4)	5 (2)	0.11	**0.041**
Receipt of allogeneic SCT	4 (0.8); n = 516	0 (0)	3 (1); n = 236	1 (0.4); n = 238	1.00	0.55
Receipt of SOT	6 (1)	1 (2)	2 (0.8)	3 (1)	0.39	0.53
T or B cell immunosuppressant drugs, other than corticosteroids ≤90 d before ICU admission	31 (6); n = 514	7 (17)	12 (5); n = 233	12 (5); n = 239	**0.014**	**0.024**
Inherited severe immunodeficiency	0 (0); n = 517	0 (0)	0 (0); n = 236	0 (0); n = 239	NA	NA
Corticosteroid use						
Systemic corticosteroids ≤30 d before ICU admission	38 (9); n = 430	7 (18); n = 39	14 (9); n = 160	17 (7); n = 231	0.14	0.12
Cumulative corticosteroid dose ≤30 d before ICU admission, mg prednisone equivalent/kg bodyweight	1.82 (1.18–3.48); n = 36	1.74 (1.28–2.37); n = 7	2.03 (0.61–3.73); n = 13	1.82 (1.42–3.60); n = 16	0.76	0.87

Characteristics	Total population, n = 519	CAPA, n = 42	CAPA excluded, n = 237	CAPA not classifiable, n = 240	p value†	p value‡
Inhalational corticosteroids ≤30 d before ICU admission	39 (10); n = 406	5 (14); n = 37	16 (11); n = 150	18 (8); n = 219	0.57	0.46
COVID-19 diagnosis						
PCR-confirmed COVID-19 (any positive PCR)	514 (99); n = 518	42 (100)	232 (98); n = 236	240 (100)	1.00	0.14
Chest x-ray or CT suggestive of COVID-19	352 (79); n = 445††	22 (69); n = 32	160 (79); n = 202	170 (81); n = 211	0.32	0.58
CT severity score at admission	15 (11–18); n = 155	15 (13–17); n = 7	16 (11–19); n = 75	15 (10–17); n = 73	0.64	0.16
Clinical data at ICU admission						
APACHE II score	14 (11–19); n = 163	20 (13–27); n = 12	16 (12–21); n = 64	13 (9–17); n = 87	0.24	**0.001**
Microbiology at admission						
Positive *Aspergillus* culture respiratory sample ≤6 mo before ICU admission	1 (0.3); n = 368	1 (3); n = 34	0 (0); n = 126	0 (0); n = 208	0.21	0.09
Positive *Aspergillus* culture respiratory sample at the time of COVID-19 diagnosis	4 (2); n = 186	2 (9); n = 22	2 (2); n = 89	0 (0); n = 75	0.18	**0.037**

*Data are presented as no. (%) or median (IQR), unless otherwise stated. Continuous variables were compared by Mann-Whitney U test, categorical variables by Fisher exact test with omission of missing data, unless stated otherwise. Percentages might not accumulate to 100% because of rounding off of numbers. Bold text indicates statistical significance. AKI, acute kidney injury APACHE, acute physiology and chronic health evaluation; BAL, broncho-alveolar lavage; BALF, broncho-alveolar lavage fluid BL, bronchial lavage; BMI, body mass index; CAPA, COVID-19–associated pulmonary aspergillosis; CKD, chronic kidney disease; COPD, chronic obstructive pulmonary disease; COVID-19, coronavirus disease; C_t, cycle threshold; CT, computed tomography; ECMO, extracorporeal membrane oxygenation; EORTC/MSGERC, European Organization for Research and Treatment of Cancer and the Mycoses Study Group Education and Research Consortium (*6*); GM, galactomannan; ICU, intensive care unit; IQR, interquartile range; LOS, length of stay; NA, not applicable; NBL, nonbronchoscopic lavage; NO, nitric oxide; RRT, renal replacement therapy; SARS-CoV-2, severe acute respiratory syndrome coronavirus 2; SCT, stem cell transplantation; SOT, solid organ transplant; TB, tuberculosis.

†CAPA versus CAPA excluded; comparisons made by Mann-Whitney U test.

‡CAPA versus CAPA excluded versus CAPA not classifiable; comparisons made by Kruskal-Wallis test.

§A patient who smoked ≤1 month before ICU admission was considered a current smoker.

¶Includes hypertension.

#Includes any use of systemic corticosteroids before ICU admission; If data ≥1 EORTC host factors were missing, then data were regarded as missing for this variable.

**Neutropenia $<0.5 \times 10^9$/L for >10 d.

††At least one thoracic X-ray or CT scan result at admission known for 445 patients.

Appendix Table 3. Laboratory results at ICU admission in the discovery cohort in a multinational observational study of COVID-19–associated pulmonary aspergillosis*

Laboratory tests	Total population, n = 519	CAPA, n = 42	CAPA excluded, n = 237	CAPA not classifiable, n = 240	p value†	p value‡
CRP, mg/L	175 (101–263); n = 447	177 (100–248); n = 34	197 (114–278); n = 200	161 (96–247); n = 213	0.327	**0.048**
Haemoglobin, mmol/L	7.6 (6.8–8.4); n = 457	7.7 (6.6–8.8); n = 34	7.5 (6.9–8.4) (n = 207)	7.7 (6.8–8.4); n = 216	0.666	0.87
Leukocytes, cells × 10^9/L	8.2 (5.9–11.3; n = 457	6.9 (5.2–10.2; n = 33	8.7 (6.4–11.6; n = 207)	7.7 (5.6–11.1); n = 217	0.061	**0.035**
Neutrophils, cells × 10^9/L	6.50 (4.48–9.24); n = 347	5.84 (4.10–7.40); n = 31	6.97 (5.00–9.39); n = 154	6.10 (4.20–9.21); n = 162	0.100	0.16
Lymphocytes, cells × 10^9/L	0.75 (0.50–1.02); n = 350	0.58 (0.45–1.02); n = 31	0.79 (0.50–1.10); n = 156	0.78 (0.51–1.00); n = 163	0.211	0.36
NLR	9.0 (5.1–14.5); n = 345	9.1 (5.1–15.1); n = 31	9.09 (5.61–14.50); n = 153	8.7 (4.5–14.3); n = 161	0.900	0.67
Platelets, cells × 10^9/L	223 (163–294); n = 456	218 (174–299); n = 33	222 (156–303); n = 207	228 (172–288); n = 216	0.989	0.89
BUN, mmol/L	7.9 (5.1–14.1); n = 442	11.7 (7.4–21.3); n = 34	7.8 (5.4–11.8); n = 199	7.5 (4.8–15.0); n = 209	**0.002**	**0.012**
Creatinine, µmol/L	81 (61–108); n = 460	84 (60–152); n = 34	81 (63–105); n = 210	81 (60–111); n = 216	0.397	0.75
Total bilirubin, µmol/L	8.2 (6.0–12.0); n = 429	8.6 (6.8–10.9); n = 33	8.8 (6.8–12.0); n = 188	8.0 (5.5–12.0); n = 208	0.533	0.23
Direct bilirubin, µmol/L	5.1 (3.5–7.4); n = 114	5.6 (4.0–9.1); n = 10	5.3 (3.8–8.6); n = 42	5.0 (3.1–6.5); n = 62	0.880	0.25
ALAT, U/L	37 (24–59); n = 432	34 (21–52); n = 33	38 (25–59); n = 193	38 (23–64); n = 206	0.143	0.32
GGT, U/L	61 (35–125); n = 407	64 (34–112); n = 31	65 (39–136); n = 178	60 (32–118); n = 198	0.591	0.29
Ferritin, µg/L	1,390 (718–2,146); n = 245	1,597 (960–3,761); n = 18	1,471 (884–2,424); n = 117	1,154 (544–1,672); n = 110	0.194	**0.002**
Albumin, g/L	29 (24–32); n = 402	28.5 (26.0–33.0); n = 31	27 (22–32); n = 190	30 (26–33); n = 181	0.061	**<0.001**
Total protein level, g/L	63 (59–68); n = 126	63 (57–66); n = 18	61 (59–65); n = 39	64 (59–70); n = 69	0.757	0.09
INR	1.2 (1.1–1.3); n = 312	1.1 (1.1–1.2); n = 27	1.2 (1.1–1.3); n = 113	1.1 (1.1–1.3); n = 172	0.724	0.90
D-dimer ≥500 ng/mL	277 (93); n = 298	25 (96); n = 26	128 (94); n = 136	124 (91); n = 136	1.000	0.66
D-dimer, ng/mL; if ≥500 ng/mL	1,720 (1,128–3,965); n = 277	2,170 (1,245–5,959); n = 25	2,135 (1,265–5,878); n = 128	1,398 (989–2,555); n = 124	0.927	**0.001**

*Data are presented as no. (%) or median (IQR), unless otherwise stated. The most strongly abnormal laboratory results obtained during the first 24 h of ICU admission are shown. Continuous variables were compared by Mann-Whitney U test or Kruskal-Wallis test, categorical variables by Fisher exact test with omission of missing data, unless stated otherwise. Percentages might not accumulate to 100% because of rounding off of numbers. Bold text indicates statistical significance. ALAT, alanine aminotransferase; BUN, blood urea nitrogen; CAPA, COVID-19–associated pulmonary aspergillosis; COVID-19, coronavirus disease; CRP, C-reactive protein; GGT, gamma glutamyl transpeptidase; ICU, intensive care unit; INR, international normalized ratio; IQR, interquartile range; NLR, neutrophil/lymphocyte ratio.
†CAPA versus CAPA excluded; comparisons made by Mann-Whitney U test.
‡CAPA versus CAPA excluded versus CAPA not classifiable; comparisons made by Kruskal-Wallis test.

Appendix Table 4. Treatment and outcome data in the discovery cohort in a multinational observational study of COVID-19–associated pulmonary aspergillosis*

Characteristics	Total population, n = 519	CAPA, n = 42	CAPA excluded, n = 237	CAPA not classifiable, n = 240	p value†	p value‡
ICU treatment data						
Invasive mechanical ventilation	423 (82); n = 517	40 (98); n = 41	225 (95)	158 (66); n = 239	0.70	**<0.0001**
Ventilation in prone position	323 (63); n = 517	26 (63); n = 41	195 (82)	102 (43); n = 239	**0.011**	**<0.0001**
Ventilation with NO	17 (3); n = 518	5 (12)	8 (3)	4 (2); n = 239	**0.031**	**0.008**
ECMO§	16 (3); n = 518	3 (7)	10 (4)	3 (1); n = 239	0.42	**0.029**
No. invasive ventilation days¶	14 (9–24); n = 395	16 (13–27); n = 37	18 (11–30); n = 212	10 (6–14); n = 146	0.98	**<0.001**
Any vasopressors and/or inotropes during ICU admission	413 (80); n = 516	41 (98)	223 (95); n = 235	149 (62); n = 239	0.70	**<0.0001**
AKI at any time during ICU admission#	264 (54); n = 486	29 (71); n = 41	137 (63); n = 218	98 (43); n = 227	0.38	**<0.0001**
AKI stage 1	89 (35); n = 258	7 (24); n = 29	44 (33); n = 135	38 (40); n = 94	0.51	0.23
AKI stage 2	47 (18); n = 258	4 (14); n = 29	31 (23); n = 135	12 (13); n = 94	0.33	0.13
AKI stage 3	122 (47); n = 258	18 (62); n = 29	60 (44); n = 135	44 (47); n = 94	0.10	0.23
RRT during ICU admission	93 (18); n = 516	17 (41)	44 (19); n = 236	32 (13); n = 238	**0.004**	**0.0004**
Systemic corticosteroids during ICU admission	216 (42); n = 516	20 (48)	131 (56); n = 236	65 (27); n = 238	0.40	**<0.0001**
Cumulative corticosteroid dose during ICU admission, mg prednisone equivalent/kg bodyweight	5.37 (1.14–13.99); n = 210	5.30 (2.36–14.30); n = 20	6.63 (1.03–16.73); n = 126	4.21 (1.22–10.80); n = 64	0.88	0.13
COVID-19 treatment data during hospital admission						
Chloroquine	162 (31)	7 (17)	114 (48)	41 (17)	**0.0001**	**<0.0001**
Hydroxychloroquine	182 (35)	21 (50)	61 (26)	100 (42)	**0.003**	**0.0001**
Remdesivir	21 (4)	1 (2)	18 (8)	2 (0.8)	0.33	**0.0004**
Lopinavir/ritonavir	20 (4)	1 (2)	6 (3)	13 (5)	1.00	0.24
Anakinra	26 (5)	0 (0)	25 (11)	1 (0.4)	**0.019**	**<0.0001**
Tocilizumab	5 (1)	1 (2)	2 (0.8)	2 (0.8)	0.39	0.59
Other	101 (20)	9 (21)	39 (17)	53 (22)	0.51	0.28
None of the above	155 (30)	14 (33)	50 (21)	91 (38)	0.11	**0.0003**
Outcome data						
ICU death	154 (30); n = 518	22 (52)	81 (34)	51 (21); n = 239	**0.036**	**<0.0001**
ICU LOS, d**	14 (8–24); n = 491	18 (12–27); n = 39	20 (12–32); n = 222	10 (5–15); n = 230	0.84	**<0.001**
ICU LOS, if alive at ICU discharge, d**	15 (9–25); n = 337	21 (16–41); n = 17	23 (13–34); n = 141	11 (6–17); n = 179	0.61	**<0.001**
Hospital LOS, d††	22 (13–35); n = 428	25 (15–38); n = 33	28 (17–45); n = 195	18 (10–27); n = 200	0.32	**<0.001**
Hospital LOS, if alive at ICU discharge, d††	26 (17–39); n = 274	34 (25–40); n = 11	36 (23–51); n = 114	20 (14–29); n = 149	0.60	**<0.001**

*Data are presented as no. (%) or median (IQR), unless otherwise stated. Continuous variables were compared by Mann-Whitney U test or Kruskal-Wallis test, categorical variables by Fisher exact test with omission of missing data, unless stated otherwise. Percentages might not accumulate to 100% because of rounding off of numbers. Bold text indicates statistical significance. AKI, acute kidney injury; CAPA, COVID-19–associated pulmonary aspergillosis; COVID-19, coronavirus disease; ECMO, extracorporeal membrane oxygenation; ECCO2R, extracorporeal CO_2 removal; ICU, intensive care unit; IQR, interquartile range; LOS, length of stay; NO; nitric oxide; RRT, renal replacement therapy.
†CAPA versus CAPA excluded; comparisons made by Mann-Whitney U test.
‡CAPA versus CAPA excluded versus CAPA not classifiable; comparisons made by Kruskal-Wallis test.
§Including one patient with Extracorporeal CO2 removal (ECCO2R).
¶If transferred to another hospital from ICU and still on ventilatory support of any kind, duration of invasive mechanical ventilatory support was regarded as missing data and not taken into account in the analyses. The same holds true for those who received a tracheostomy for a prolonged weaning trajectory.
#Acute kidney injury definition and staging according to Kidney Disease: Improving Global Outcomes (KDIGO; https://kdigo.org) criteria.
**Data on ICU LOS were regarded as missing if transfer to another hospital was the reason for ICU discharge.
††Data on hospital LOS were regarded as missing if patients deceased after ICU discharge because no data were available whether or not death occurred in hospital.

Appendix Table 5. CAPA diagnosis among participating centers in a multinational observational study of COVID-19–associated pulmonary aspergillosis*

Participating center	CAPA	CAPA excluded	CAPA not classifiable	CAPA in classifiable patients
Discovery cohort				
Amsterdam University Medical Centers, Academisch Medisch Centrum (AMC), Amsterdam, the Netherlands	7/80 (9)	32/80 (40)	41/80 (51)	7/39 (18)
Amsterdam University Medical Centers, Vrije Universiteit Medisch Centrum (VUmc), Amsterdam, the Netherlands	7/69 (10)	39/69 (57)	23/69 (33)	7/46 (15)
Erasmus Medical Center, Rotterdam, the Netherlands	3/85 (4)	22/85 (26)	60/85 (71)	3/25 (12)
Radboud University Medical Center, Nijmegen, the Netherlands	3/80 (4)	77/80 (96)	0/80 (0)	3/80 (4)
Ziekenhuis Netwerk Antwerpen (ZNA) Campus Stuivenberg, Antwerpen, Belgium	10/41 (24)	15/41 (37)	16/41 (39)	10/25 (40)
Algemeen Ziekenhuis (AZ) St-Jan Brugge_Oostende, Brugge, Belgium	1/14 (7)	7/14 (50)	6/14 (43)	1/8 (13)
University Hospitals Leuven, Leuven, Belgium	9/92 (10)	33/92 (36)	50/92 (54)	9/42 (21)
Algemeen Ziekenhuis (AZ) Delta Hospital, Roeselare, Belgium	2/58 (3)	12/58 (21)	44/58 (76)	2/14 (14)
Validation cohort				
Amiens University Hospital, Amiens, France	13/119 (11)	75/119 (63)	31/119 (26)	13/88 (15)
Lille University Hospital, Lille, France	8/128 (6)	101/128 (79)	19/128 (15)	8/109 (7)
Rouen University Hospital, Rouen, France	0/57 (0)	8/57 (14)	49/57 (86)	0/8 (0)

*Results are reported as no. patients/no. patients included per center (%). CAPA classification according to the European Confederation for Medical Mycology/International Society for Human and Animal Mycology (ECMM/ISHAM) classification (*1*). CAPA, COVID-19–associated pulmonary aspergillosis; COVID-19, coronavirus disease.

Appendix Table 6. Application of different classification systems in the discovery cohort in a multinational observational study of COVID-19–associated pulmonary aspergillosis*

Classification	Physician reported, n = 45	ECMM/ISHAM proven or probable CAPA, n = 38†,‡	IAPA expert case definition, set A, proven or probable IPA, n = 37‡	m*Asp*ICU proven or putative IPA, n = 31‡,§	EORTC/MSGERC proven or probable IPA, n = 20‡,§
Physician reported, n = 45		32	31	24	14
ECMM/ISHAM proven or probable CAPA, n = 38†,‡	32		37	26	16
IAPA expert case definition, set A, proven or probable IPA, n = 37‡	31	37		26	16
m*Asp*ICU proven or putative IPA, n = 31‡,§	24	26	26		19
EORTC/MSGERC proven or probable IPA, n = 20‡,§	14	16	16	19	

*CAPA, COVID-19–associated pulmonary aspergillosis; COVID-19, coronavirus disease; ECMM/ISHAM, European Confederation for Medical Mycology/International Society for Human and Animal Mycology; EORTC/MSGERC, European Organization for Research and Treatment of Cancer and the Mycoses Study Group Education and Research Consortium; IAPA, influenza-associated pulmonary aspergillosis; IPA, invasive pulmonary aspergillosis; m*Asp*ICU, modified *Aspergillus* intensive care unit classification.
†European Confederation for Medical Mycology/International Society for Human and Animal Mycology (ECMM/ISHAM) classification (*1*).
‡Radiological findings were not considered for any of the classification systems; therefore, for the IAPA expert case definition classification, only subset A for probable pulmonary IPA, which does not require cavitating infiltrates specifically on chest radiography, could be assessed.
§For the m*Asp*ICU (*4*,*5*) and EORTC/MSGERC classifications (*6*), clinical factors were not taken into account. For the m*Asp*ICU classification, all lower respiratory tract cultures positive for *Aspergillus*, including bronchial lavage (BL), broncho-alveolar lavage (BAL) and mini-BAL fluid cultures, were considered as a positive entry criterion.

Appendix Table 7. Mycological diagnostic test results according to diagnostic group in the discovery cohort in a multinational observational study of COVID-19–associated pulmonary aspergillosis*

Characteristics	Total population, n = 519	CAPA, n = 42	CAPA excluded, n = 237	CAPA not classifiable, n = 240	p value†	p value‡
Proven CAPA	6 (2); n = 279	6 (14)	NA	NA	NA	NA
Histological examination of biopsy	5 (83); n = 6	5 (83); n = 6	NA	NA	NA	NA
Autopsy indicative of CAPA	2 (33); n = 6	2 (33); n = 6	NA	NA	NA	NA
Serum GM						
Serum GM performed	176 (34)	28 (67)	148 (62)	0 (0)	0.73	**<0.0001**
>1 Serum GM performed on separate days	95 (71); n = 134	14 (50); n = 28	81 (76); n = 106	NA	**0.010**	**0.010**
Serum GM OD >0.5	3 (2); n = 176§	3 (11); n = 28	0 (0); n = 148	NA	NA	NA
Serum GM OD >0.5 on multiple, separate days	0 (0); n = 3	0 (0); n = 3	NA	NA	NA	NA
Serum GM OD						
All¶	0.10 (0.10–0.10); n = 134	0.10 (0.06–0.14); n = 28	0.10 (0.10–0.10); n = 106	NA	0.95	0.95
If OD >0.5¶	1.00 (0.80–..); n = 3	1.00 (0.80–..); n = 3	NA	NA	NA	NA
Days between ICU admission and first reported serum GM#	5 (3–8); n = 133	7 (5–14); n = 27	5 (3–7); n = 106	NA	**0.005**	**0.005**
Days between ICU admission and first reported serum GM OD >0.5#	15 (4–..); n = 3	15 (4–..); n = 3	NA	NA	NA	NA
BAL/BL						
Any performed/reported	166 (32)	41 (98)	125 (53)	NA	**<0.0001**	**<0.0001**
Positive BALF/BL culture	17 (10); n = 166	17 (42); n = 41	0 (0); n = 125	NA	NA	NA
BALF/BL GM obtained on multiple, separate days	33 (37); n = 90	16 (46); n = 35	17 (31); n = 55	NA	0.18	0.18
BALF/BL GM OD ≥1.0	32 (19); n = 166**	32 (78); n = 41††	0 (0); n = 125‡‡	NA	NA	NA
BALF/BL GM OD ≥1.0 on multiple, separate days	8 (25); n = 32	8 (25); n = 32	NA	NA	NA	NA
BALF/BL GM OD value						
All¶	0.20 (0.10–1.50); n = 90	1.80 (1.00–3.90); n = 35	0.10 (0.10–0.20); n = 55	NA	**<0.001**	**<0.001**
If OD ≥1.0¶	2.72 (1.75–4.00); n = 32	2.72 (1.75–4.00); n = 32	NA	NA	NA	NA
Days between ICU admission and first reported BALF/BL GM#	9 (5–14); n = 88	7 (4–14); n = 33	10 (5–14); n = 55	NA	0.21	0.21
Days between ICU admission and first reported BALF/BL GM OD ≥1.0#	7 (5–13); n = 30	7 (5–13); n = 30	NA	NA	NA	NA
Positive BALF/BL PCR, any C_t value	9 (5); n = 166§§	7 (17); n = 41¶¶	2 (2); n = 125##	NA	NA	NA
NBL						
Any performed/reported	42 (8)	9 (21)	30 (13)	3 (1)	0.15	**<0.0001**
Positive NBL culture	6 (14); n = 42	6 (67); n = 9	0 (0); n = 30	0 (0); n = 3	NA	NA
Positive plasma, serum, or whole blood PCR	ND	ND	ND	ND	NA	NA
1 NBL GM OD >4.5	5 (12); n = 42	5 (56); n = 9	0 (0); n = 30	0 (0); n = 3	NA	NA
≥2 NBL GM OD >1.2	0 (0); n = 7***	0 (0); n = 3	0 (0); n = 4	0 (0); n = 0	NA	NA
NBL GM OD >1.2 and positive PCR	5 (56); n = 9†††	5 (83); n = 6	0 (0); n = 3	0 (0); n = 0	NA	NA
Time to first positive mycological test						
Days between ICU admission and first positive mycological test‡‡‡	NA	6 (3–9); n = 41	NA	NA	NA	NA

*Data are presented as no. (%) or median (IQR), unless otherwise stated. Continuous variables were compared by Mann-Whitney U test or Kruskal-Wallis test, categorical variables by Fisher exact test with omission of missing data, unless stated otherwise. Percentages might not accumulate to 100% because of rounding off of numbers. Bold text indicates statistical significance. BAL, bronchoalveolar lavage; BALF, bronchoalveolar lavage fluid; BL, bronchial lavage; BMI, body mass index; CAPA, COVID-19–associated pulmonary aspergillosis; COVID-19, coronavirus disease; C_t, cycle threshold; GM, galactomannan; ICU, intensive care unit; IQR, interquartile range; ND, not done; NA, not applicable; OD, optical density.
†CAPA versus CAPA excluded; comparisons made by Mann-Whitney U test.
‡CAPA versus CAPA excluded versus CAPA not classifiable; comparisons made by Kruskal-Wallis test.
§Serum GM values available for 134 patients.

Characteristics	Total population, n = 519	CAPA, n = 42	CAPA excluded, n = 237	CAPA not classifiable, n = 240	p value†	p value‡

¶When multiple values were reported for one patient, the median of these values was used for further calculations.
#Dates were regarded as missing if inconsistencies regarding date of obtaining test results existed.
**BALF/BL GM OD values reported for 90 patients, but 166 patients underwent BALF/BL sampling.
††BALF/BL GM OD values reported for 35 patients.
‡‡BALF/BL GM OD values reported for 55 patients.
§§BALF/BL PCR results reported for 11 patients.
¶¶BALF/BL PCR results reported for 7 patients.
BALF/BL PCR results reported for 4 patients. Positive PCR with C_t values ≥36 as only positive mycological criterion.
***Multiple NBL GM OD values reported for 7 patients.
†††Results for both NBL GM and PCR reported in 9 patients.
‡‡‡ Mycological test considered a criterion for proven, probable, or possible CAPA according to the 2020 ECMM/ISHAM classification.

Appendix Table 8. Antifungal treatment in the discovery and validation cohorts in a multinational observational study of COVID-19–associated pulmonary aspergillosis*

Cohort and antifungal treatment	Total population	CAPA	CAPA excluded	CAPA not classifiable
Discovery cohort	N = 519	N = 42	N = 237	N = 240
Any antifungal treatment during ICU admission	83 (16)	37 (88)	36 (15)	10 (4)
Initial antifungal treatment	N = 83	N = 37	N = 36	N = 10
Voriconazole	24 (29)	20 (54)	3 (8)	1 (10)
Other azole monotherapy	13 (16)	2 (5)	3 (8)	8 (80)
Echinocandin monotherapy	8 (10)	1 (3)	7 (19)	0 (0)
Azole + echinocandin combination therapy	33 (40)	13 (35)	19 (53)	1 (10)
Liposomal amphotericin B monotherapy	2 (2)	1 (3)	1 (3)	0 (0)
Other combination therapy	1 (1)	0 (0)	1 (3)	0 (0)
Unspecified	2 (2)	0 (0)	2 (6)	0 (0)
Validation cohort	N = 304	N = 21	N = 188	N = 95
Any antifungal treatment used	57 (19)	12 (57)	37 (20)	8 (8)
Specified antifungal treatment	N = 57	N = 12	N = 37	N = 8
Voriconazole	14 (25)	7 (58)	7 (19)	0 (0)
Isavuconazole	3 (5)	3 (25)	0 (0)	0 (0)
Fluconazole	11 (19)	0 (0)	8 (22)	3 (38)
Caspofungin	21 (37)	1 (8)	18 (49)	2 (25)
Amphotericin B	3 (5)	2 (17)	1 (3)	0 (0)
Combination of isavuconazole and liposomal amphotericin B	1 (2)	1 (8)	0 (0)	0 (0)
Unspecified antifungal treatment	8 (14)	0 (0)	5 (14)	3 (38)

*Data are presented as no. (%) and for the discovery cohort include antifungal treatment administered in ICU, including treatment initiated before ICU admission, but continued in ICU. CAPA, COVID-19–associated pulmonary aspergillosis; COVID-19, coronavirus disease; ICU, intensive care unit.

Appendix Table 9. Demographic and clinical characteristics of the validation cohort in a multinational observational study of COVID-19–associated pulmonary aspergillosis*

Characteristics	Total population, n = 304	CAPA, n = 21	CAPA excluded, n = 188	Not classifiable, n = 95	p value†	p value‡
Age, y	63 (55–71)	67 (59–75)	62 (53–69)	64 (56–72)	0.06	0.1
Sex						
F	227 (75)	21 (100)	141 (75)	65 (68)		
M	77 (25)	0 (0)	47 (25)	30 (32)	**0.005**	**0.004**
BMI, kg/m^2	30.0 (26.0–34.4); n = 296	30.2 (26.1–32.8); n = 20	30.0 (26.4–34.5); n = 185	29.4 (25.4–36.1); n = 91	0.84	0.77
BMI >30 kg/m^2	142 (48); n = 296	10 (50); n = 20	90 (49); n = 185	42 (46); n = 91	1.00	0.89
Smoking status						
Current smoker	10 (3); n = 295	0 (0)	7 (4); n = 180	3 (3); n = 94	1.00	1.00
Not current smoker	285 (97); n = 295	21 (100)	173 (96); n = 180	91 (97); n = 94	1.00	1.00
Unknown	9 (3)	0 (0)	8 (4)	1 (1)	1.00	0.37
Underlying conditions						
Active hematological malignancy	10 (3)	0 (0)	6 (3)	4 (4)	1.00	0.87
Hypertension	165 (54)	15 (71)	96 (51)	54 (57)	0.106	0.18
Cardiovascular disease§	64 (21)	9 (43)	35 (19)	20 (21)	**0.020**	**0.046**
Cardiovascular disease, including hypertension§	185 (61)	17 (81)	112 (60)	56 (59)	0.06	0.15
Diabetes mellitus	92 (30)	9 (43)	62 (33)	21 (22)	0.47	0.07
Asthma	22 (7)	2 (10)	12 (6)	8 (8)	0.64	0.67
COPD	20 (7)	2 (10)	12 (6)	6 (6)	0.64	0.80
Liver cirrhosis¶	5 (2)	2 (10)	2 (1)	1 (1)	0.051	**0.046**
Autoimmune disease	16 (5)	2 (10)	11 (6)	3 (3)	0.63	0.32
HIV/AIDS	3 (1)	0 (0)	1 (0.5)	2 (2)	1.00	0.41

Characteristics	Total population, n = 304	CAPA, n = 21	CAPA excluded, n = 188	Not classifiable, n = 95	p value†	p value‡
Active solid organ malignancy	4 (1)	1 (5)	3 (2)	0 (0)	0.35	0.16
CKD	18 (6)	3 (14)	10 (5)	5 (5)	0.13	0.25
Sleep apnea syndrome	36 (12)	2 (10)	21 (11)	13 (14)	1.00	0.81
Bronchiectasis	5 (2)	2 (10)	1 (0.5)	2 (2)	**0.027**	**0.018**
EORTC/MSGERC host factors						
Any#	35 (12)	3 (14)	20 (11)	12 (13)	0.71	0.74
Recent neutropenia**	0 (0); n = 303	0 (0)	0 (0); n = 187	0 (0)	NA	NA
Hematological malignancy	10 (3)	0 (0)	6 (3)	4 (4)	1.00	0.87
Receipt of SOT	9 (3)	1 (5)	5 (3)	3 (3)	0.48	0.63
Corticosteroids ≥0.3 mg/kg for ≥3 wks within the past 60 d	17 (6)	2 (10)	10 (5)	5 (5)	0.34	0.61
Other immunosuppressant drugs ≤90 d before ICU admission	23 (8)	2 (10)	16 (9)	5 (5)	0.70	0.60
Clinical data at ICU admission						
SAPS II score	41 (30–58); n = 285	48 (36–68); n = 17	44 (32–63); n = 185	35 (24–46); n = 83	0.38	**<0.001**
ARDS††	212 (70); n = 303	17 (85); n = 20	150 (80)	45 (47)	0.77	**<0.001**

*Data are presented as no. (%) or median (IQR), unless otherwise stated. Continuous variables were compared by Mann-Whitney U test or Kruskal-Wallis test, categorical variables by Fisher exact test with omission of missing data, unless stated otherwise. Percentages might not accumulate to 100% because of rounding off of numbers. Bold text indicates statistical significance. APACHE, acute physiology and chronic health evaluation; ARDS, acute respiratory distress syndrome; BMI, body mass index; CAPA, COVID-19–associated pulmonary aspergillosis; CKD, chronic kidney disease; COPD, chronic obstructive pulmonary disease; COVID-19, coronavirus disease; EORTC/MSGERC, European Organization for Research and Treatment of Cancer and the Mycoses Study Group Education and Research Consortium (*6*); ICU, intensive care unit; IQR, interquartile range, NA, not applicable; SAPS, simplified acute physiology score; SOT, solid organ transplant.
†CAPA versus CAPA excluded; comparisons made by Mann-Whitney U test.
‡CAPA versus CAPA excluded versus CAPA not classifiable; comparisons made by Kruskal-Wallis test.
§Includes Factor V Leiden mutation.
#Includes any use of systemic corticosteroids We did not assess receipt of an allogeneic stem cell transplant, presence of an inherited severe immunodeficiency, and presence of acute graft-versus-host disease.
**Neutropenia <0.5 × 10^9/L for >10 d.
††ARDS according to the Berlin definition.

Appendix Table 10. Treatment and outcome data in the validation cohort in a multinational observational study of COVID-19–associated pulmonary aspergillosis*

Characteristics	Total population; n = 304	CAPA; n = 21	CAPA excluded; n = 188	Not classifiable; n = 95	p value†	p value‡
ICU treatment data						
Invasive mechanical ventilation	228 (76); n = 302	19 (95); n = 20	168 (89)	41 (44); n = 94	0.70	**<0.0001**
Ventilation in prone position	159 (53); n = 302	15 (75); n = 20	116 (62)	28 (30); n = 94	0.33	**<0.0001**
ECMO	41 (14); n = 303	4 (20); n = 20	34 (18)	3 (3)	0.77	**0.0004**
No. invasive ventilation days	15 (9–25); n = 212	18 (13–25); n = 17	15 (9–25); n = 157	14 (10–25); n = 38	0.21	0.44
Any vasopressors and/or inotropes	189 (63); n = 302	16 (80); n = 20	138 (74); n = 187	35 (37)	0.79	**<0.0001**
AKI at any time during ICU admission§	126 (42); n = 303	16 (80); n = 20	94 (50)	16 (17)	**0.017**	**<0.0001**
RRT	64 (21); n = 303	11 (55); n = 20	47 (25)	6 (6)	**0.008**	**<0.0001**
Systemic corticosteroids during ICU admission	147 (49); n = 303	11 (52)	106 (57); n = 187	30 (32)	0.82	**0.0003**
COVID-19 treatment data						
Hydroxychloroquine	56 (18)	2 (10)	35 (19)	19 (20)	0.38	0.59
Azithromycin	63 (21)	3 (14)	43 (23)	17 (18)	0.58	0.55
Remdesivir	10 (3)	0 (0)	9 (5)	1 (1)	0.60	0.29
Lopinavir/ritonavir	115 (38)	6 (29)	68 (36)	41 (43)	0.63	0.37
Tocilizumab	13 (4)	2 (10)	6 (3)	5 (5)	0.19	0.24
Interferon β	12 (4)	0 (0)	11 (6)	1 (1)	0.61	0.11
Oseltamivir	20 (7)	3 (14)	15 (8)	2 (2)	0.40	**0.037**
Outcome data						
Candidemia	4 (1)	0 (0)	4 (2)	0 (0)	1.00	0.48
ICU mortality rate	69 (23); n = 299	9 (43)	46 (25); n = 185	14 (15); n = 93	0.12	**0.017**
30-Day mortality rate	56 (19); n = 301	8 (38)	37 (20); n = 186	11 (12); n = 94	0.09	**0.015**
90-Day mortality rate	69 (23); n = 301	9 (43)	46 (25); n = 186	14 (15); n = 94	0.11	**0.016**
ICU LOS, d¶	14 (8–26); n = 295	22 (12–35); n = 20	18 (10–28); n = 183	9 (5–15); n = 92	0.27	**<0.001**
ICU LOS if alive at ICU discharge, d¶	14 (8–26); n = 230	30 (14–40); n = 12	18 (10–28); n = 139	8 (5–15); n = 79	0.13	**<0.001**

*Data are presented as no. (%) or median (IQR), unless otherwise stated. Continuous variables were compared by Mann-Whitney U test or Kruskal-Wallis test, categorical variables by Fisher exact test with omission of missing data, unless stated otherwise. Percentages might not accumulate to 100% because of rounding off of numbers. Bold text indicates statistical significance. AKI, acute kidney injury; CAPA, COVID-19–associated pulmonary aspergillosis; COVID-19, coronavirus disease; ECMO, extracorporeal membrane oxygenation; ICU, intensive care unit; IQR, interquartile range; LOS, length of stay; RRT, renal replacement therapy.
†CAPA versus CAPA excluded; comparisons made by Mann-Whitney U test.
‡CAPA versus CAPA excluded versus CAPA not classifiable; comparisons made by Kruskal-Wallis test.
§Acute kidney injury definition and staging according to Kidney Disease: Improving Global Outcomes (KDIGO; https://kdigo.org) criteria.
¶Data on ICU LOS were regarded as missing if still admitted at the time of data entry or if transfer to another hospital was the reason for ICU discharge.

Appendix Table 11. Application of different classification systems in the validation cohort in a multinational observational study of COVID-19–associated pulmonary aspergillosis*

Classification	Physician reported, n = 20	ECMM/ISHAM probable CAPA, n = 21†,‡	IAPA expert case definition, set A, probable IA, n = 19‡	m*Asp*ICU putative IPA, n = 13‡,§	EORTC/MSGERC probable IPA, n = 3‡,§
Physician reported, n = 20		19	19	11	2
ECMM/ISHAM probable CAPA, n = 21†,‡	19		19	11	2
IAPA expert case definition, set A, proven or probable IPA, n = 19‡	19	19		11	2
m*Asp*ICU putative IPA, n = 13‡,§	11	11	11		3
EORTC/MSGERC probable IPA, n = 3‡,§	2	2	2	3	

*CAPA, COVID-19–associated pulmonary aspergillosis; COVID-19, coronavirus disease; ECMM/ISHAM, European Confederation for Medical Mycology/International Society for Human and Animal Mycology; EORTC/MSGERC, European Organization for Research and Treatment of Cancer and the Mycoses Study Group Education and Research Consortium; IAPA, influenza-associated pulmonary aspergillosis; IPA, invasive pulmonary aspergillosis; m*Asp*ICU, modified *Aspergillus* intensive care unit classification.
†European Confederation for Medical Mycology/International Society for Human and Animal Mycology (ECMM/ISHAM) classification (*1*).
‡Radiological findings were not considered for any of the classification systems; therefore, for the IAPA expert case definition classification, only subset A for probable pulmonary IPA, which does not require cavitating infiltrates specifically on chest radiography, could be assessed.
§For the m*Asp*ICU (*4*,5) and EORTC/MSGERC classifications (*6*), clinical factors were not taken into account. For the m*Asp*ICU classification, all lower respiratory tract cultures positive for *Aspergillus*, including broncho-alveolar lavage (BAL) fluid cultures, were considered as a positive entry criterion.

Appendix Table 12. Mycological diagnostic test results according to diagnostic group in the validation cohort in a multinational observational study of COVID-19–associated pulmonary aspergillosis*

Characteristics	Total population, n = 304	CAPA, n = 21	CAPA excluded, n = 188	Not classifiable, n = 95	p value†	p value‡
Serum GM						
Serum GM performed	173 (57)	18 (86)	155 (82)	0 (0)	NA	NA
Serum GM OD >0.5	4 (2); n = 172§	4 (22); n = 18	0 (0); n = 154¶	NA	NA	NA
Serum GM OD						
All	0.07 (0.04–0.12); n = 172§	0.10 (0.06–0.34); n = 18	0.06 (0.04–0.11); n = 154¶	NA	**0.008**	**0.008**
If OD >0.5	0.66 (0.54–3.84); n = 4	0.66 (0.54–3.84); n = 4	NA	NA	NA	NA
BAL						
BAL performed/reported	135 (44)	21 (100)	114 (61)	0 (0)	NA	NA
Positive BALF culture	11 (8); n = 135	11 (52)	0 (0); n = 114	NA	NA	NA
BALF GM performed	123 (91); n = 135	21 (100)	102 (89); n = 114	NA	NA	NA
BALF GM OD ≥1.0	13 (11); n = 123	13 (62)	0 (0); n = 102	NA	NA	NA
BALF GM OD value						
All#	0.12 (0.05–0.32); n = 123	1.10 (0.12–3.06)	0.11 (0.05–0.18); n = 102	NA	**<0.001**	**<0.001**
If OD ≥1.0#	1.70 (1.24–5.38); n = 13	1.70 (1.24–5.38); n = 13	NA	NA	NA	NA
Positive BALF PCR, any C_t value	8 (13); n = 64	8 (53); n = 15	0 (0); n = 49	NA	NA	NA
Serum β-D-glucan						
Serum β-D-glucan performed	184 (61)	19 (91)	160 (85)	5 (5)	0.75	**<0.0001**
Serum β-D-glucan ≥80 pg/mL	37 (20); n = 184	8 (42); n = 19	29 (18); n =160	0 (0); n = 5	**0.030**	**0.038**
Serum β-D-glucan, pg/mL**						
All	31 (13–60); n = 184	34 (31–156); n = 19	31 (10–59); n = 160	31 (16–46); n = 5	0.055	0.15
If value ≥80 pg/mL	171 (120–288); n = 37	180 (116–316); n = 8	171 (130–283); n = 29	NA	1.00	0.99
Other mycological tests						
TBA performed	131 (43)	8 (38)	94 (50)	29 (31)	0.36	**0.006**
Positive TBA culture	7 (5); n = 131	2 (25); n = 8	5 (5); n = 94	0 (0); n = 29	0.09	0.05
Positive TBA PCR	3 (18); n = 17	0 (0); n = 1	3 (43); n = 7	0 (0); n = 9	1.00	0.08
Sputum obtained	34 (11)	3 (14)	21 (11)	10 (11)	0.72	0.84
Positive sputum culture	0 (0); n = 34	0 (0); n = 3	0 (0); n = 21	0 (0); n = 10	NA	NA
Positive sputum PCR	2 (29); n = 7	0 (0); n = 0	2 (67); n = 3	0 (0); n = 4	NA	0.14
Positive plasma, serum, or whole blood PCR	ND	ND	ND	ND	NA	NA

*Data are presented as no. (%) or median (IQR), unless stated otherwise. Continuous variables were compared by Mann-Whitney U test or Kruskal-Wallis test, categorical variables by Fisher exact test with omission of missing data, unless stated otherwise. Percentages might not accumulate to 100% because of rounding off of numbers. Bold text indicates statistical significance. BAL, broncho-alveolar lavage; BALF, BAL fluid; CAPA, COVID-19–associated pulmonary aspergillosis; COVID-19, coronavirus disease; Ct, cycle threshold; GM, galactomannan; ICU, intensive care unit; IQR, interquartile range; NA, not applicable; ND, not done; OD, optical density; TBA, tracheobronchial aspirate.
†CAPA versus CAPA excluded; comparisons made by Mann-Whitney U test.
‡CAPA versus CAPA excluded versus CAPA not classifiable; comparisons made by Kruskal-Wallis test.
§Serum GM performed in 173 patients, including 1 patient with an unknown result.
¶Serum GM OD values known for 154 patients, unknown value in 1 patient.
#One value of >6.0 entered as 6.0.
**One value of >500 pg/mL entered as 500 pg/mL.

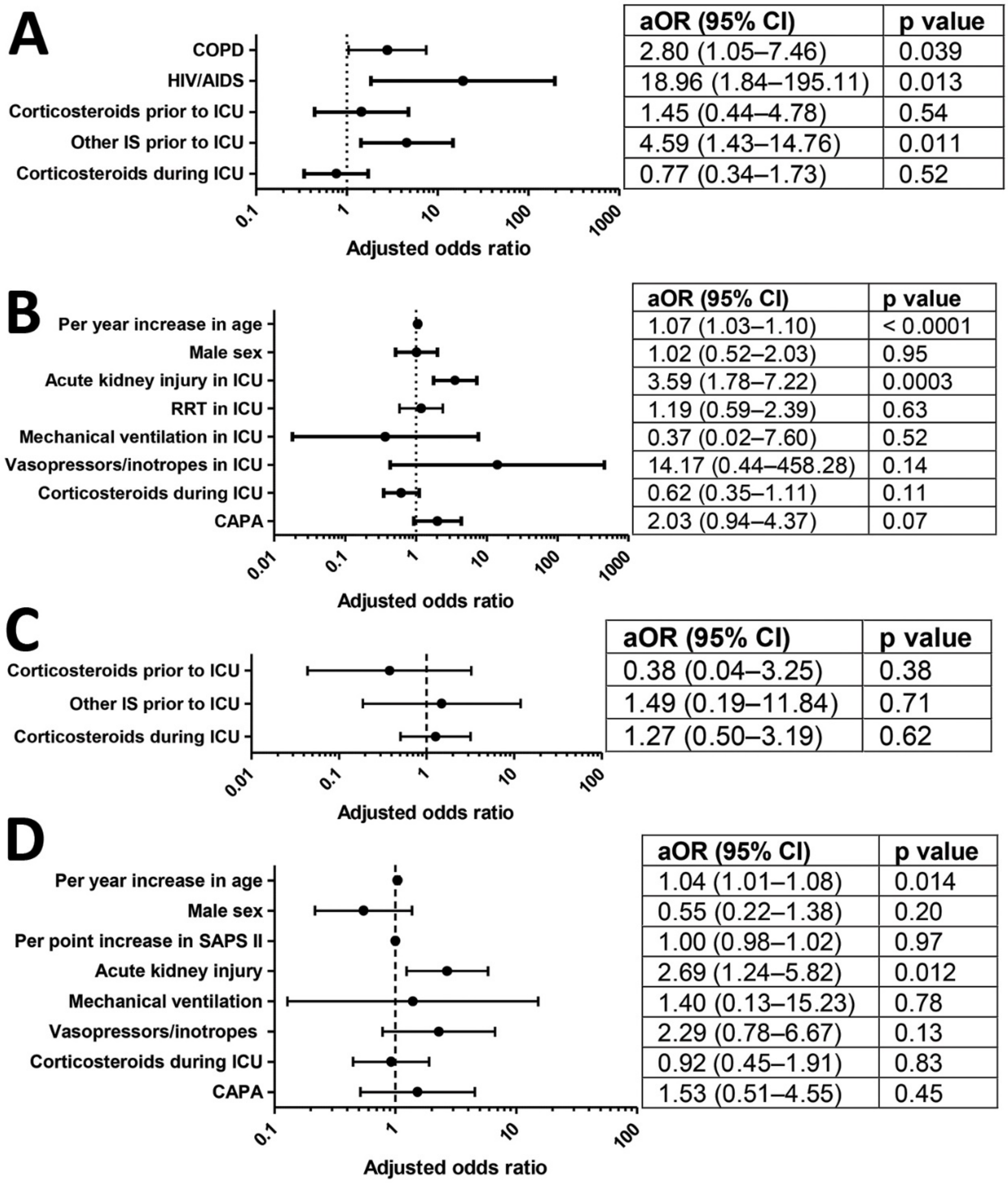

A

aOR (95% CI)	p value
2.80 (1.05–7.46)	0.039
18.96 (1.84–195.11)	0.013
1.45 (0.44–4.78)	0.54
4.59 (1.43–14.76)	0.011
0.77 (0.34–1.73)	0.52

B

aOR (95% CI)	p value
1.07 (1.03–1.10)	< 0.0001
1.02 (0.52–2.03)	0.95
3.59 (1.78–7.22)	0.0003
1.19 (0.59–2.39)	0.63
0.37 (0.02–7.60)	0.52
14.17 (0.44–458.28)	0.14
0.62 (0.35–1.11)	0.11
2.03 (0.94–4.37)	0.07

C

aOR (95% CI)	p value
0.38 (0.04–3.25)	0.38
1.49 (0.19–11.84)	0.71
1.27 (0.50–3.19)	0.62

D

aOR (95% CI)	p value
1.04 (1.01–1.08)	0.014
0.55 (0.22–1.38)	0.20
1.00 (0.98–1.02)	0.97
2.69 (1.24–5.82)	0.012
1.40 (0.13–15.23)	0.78
2.29 (0.78–6.67)	0.13
0.92 (0.45–1.91)	0.83
1.53 (0.51–4.55)	0.45

Appendix Figure 1. Forest plots of risk factors for the development of COVID-19–associated pulmonary aspergillosis (CAPA) and intensive care unit (ICU) death in a multinational observational study of CAPA. A) Binary logistic regression analysis of risk factors for the development of CAPA in the discovery cohort. B) Binary logistic regression analysis of risk factors for ICU death in the discovery cohort. C) Binary logistic regression analysis of risk factors for the development of CAPA in the validation cohort. D) Binary logistic regression analysis of risk factors for ICU death in the validation cohort. Error bars indicate 95% CI; dots indicate aOR. Dotted vertical lines indicate an aOR of 1. aOR, adjusted odds ratio; 95% CI COVID-19, coronavirus disease; COPD, Chronic obstructive pulmonary disease; IS, immunosuppressant drugs; RRT, renal replacement therapy; SAPS, simplified acute physiology score.

A

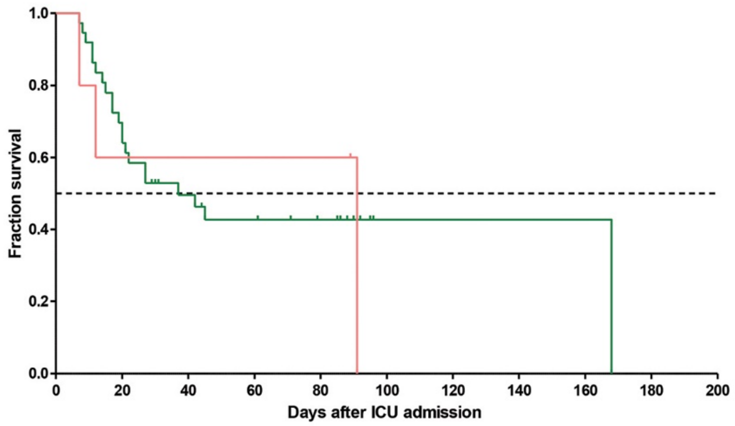

Number at risk									
CAPA, AF	37	25	16	13	10	2	2	2	2
CAPA, no AF	5	4	4	4	4	1	1	1	1

B

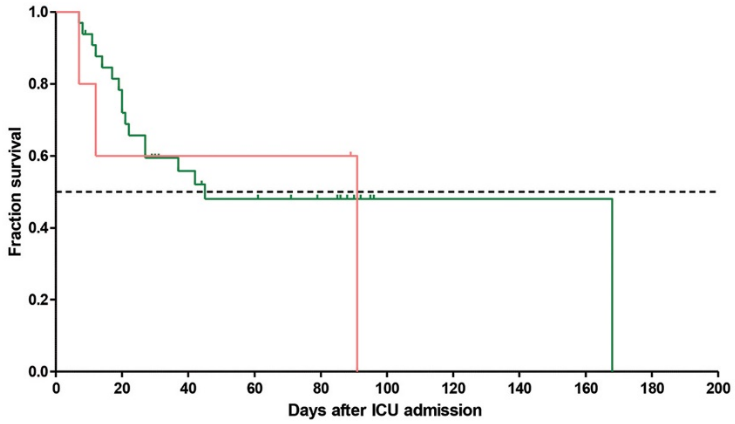

Number at risk										
CAPA, AF	33	25	16	13	10	2	2	2	2	1
CAPA, no AF	5	4	4	4	4	1	1	1	1	1

C

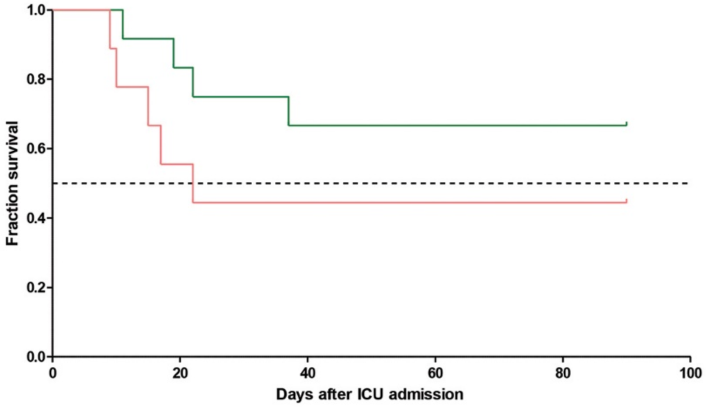

Number at risk						
CAPA, AF	12	11	9	9	9	8
CAPA, no AF	9	6	5	5	5	4

Appendix Figure 2. Kaplan-Meier survival curves comparing CAPA patients who did and did not receive any antifungal (AF) treatment during intensive care unit (ICU) admission in a multinational observational study of COVID-19–associated pulmonary aspergillosis (CAPA). Green indicates CAPA patients receiving

AF; salmon indicates patients not receiving AF. Tables show the number of patients at risk for ICU death per time point after ICU admission. Dashed horizontal lines indicate a survival fraction of 0.5. A) Discovery cohort (n = 42); (p = 0.869); B) discovery cohort after designating patients with possible CAPA to the CAPA excluded group (n = 38) (p = 0.683); C) validation cohort (n = 21) (p = 0.212). Survival analysis was performed by Mantel-Cox log rank test. COVID-19, coronavirus disease.

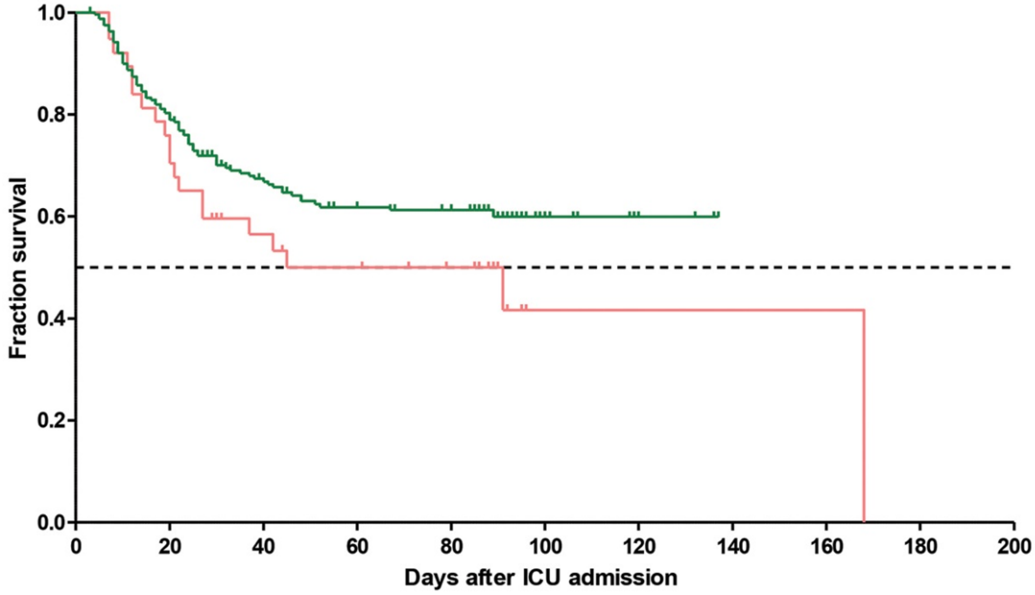

Number at risk										
CAPA	38	28	19	16	13	2	2	2	2	1
CAPA excluded	241	187	124	107	101	10	4	1	1	1

Appendix Figure 3. Kaplan-Meier survival curves comparing patients in a multinational observational study COVID-19–associated pulmonary aspergillosis (CAPA). The graph compares patients classified with CAPA (salmon) with CAPA excluded patients (green) when possible CAPA was classified as CAPA excluded (n = 279). Survival analysis was calculated by Mantel-Cox log rank test and shows survival over time is no longer significantly different within the discovery cohort when patients with possible CAPA are designated to the CAPA excluded group (p = 0.134). Median estimated survival in the CAPA group is 45 days. Tables show the number of patients at risk for ICU death per time point after ICU admission. Dashed horizontal lines indicate a survival fraction of 0.5. COVID-19, coronavirus disease.

A

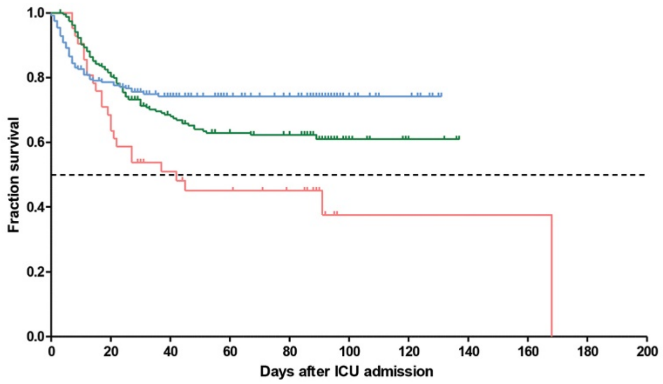

Number at risk										
CAPA	42	28	19	16	13	2	2	2	2	1
CAPA excluded	237	187	124	107	101	10	4	1	1	1
CAPA not classifiable	240	167	94	77	65	17	11	2	2	2

B

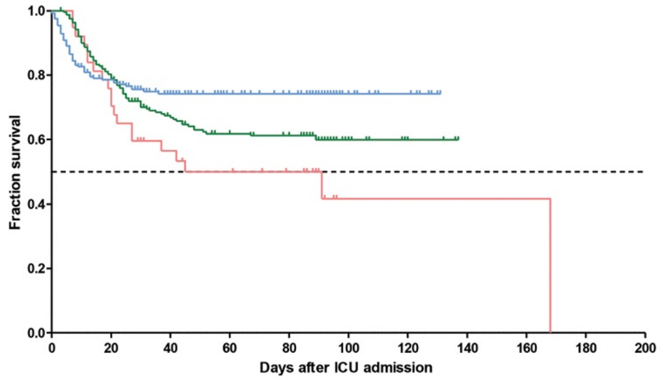

Number at risk										
CAPA	38	28	19	16	13	2	2	2	2	1
CAPA excluded	241	187	124	107	101	10	4	1	1	1
CAPA not classifiable	240	167	94	77	65	17	11	2	2	2

C

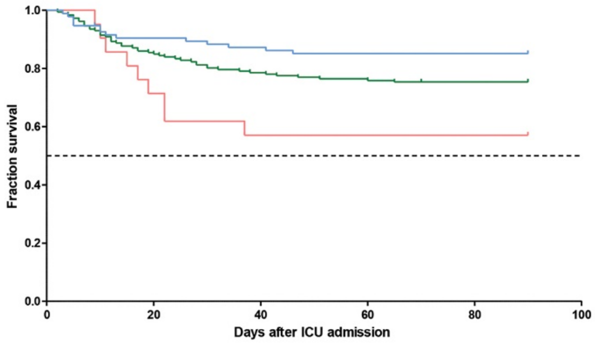

Number at risk						
CAPA	21	16	13	13	13	12
CAPA excluded	188	160	148	143	141	140
CAPA not classifiable	95	86	83	81	81	80

Appendix Figure 4. Kaplan-Meier survival curves comparing patients in a multinational observational study of COVID-19–associated pulmonary aspergillosis (CAPA). The graph compares patients classified with CAPA (salmon), CAPA excluded (green) and CAPA not classifiable (blue). Survival analysis was performed by Mantel-Cox log rank test. A) Discovery cohort (n = 519); survival over time is significantly different between the 3 groups in this cohort (p = 0.007). B) Discovery cohort after designating possible

CAPA patients to the CAPA excluded group (n = 519), survival over time remained significantly different between the 3 groups (p = 0.040). C) Validation cohort (n = 304); survival over time is significantly different between the 3 patient groups (p = 0.014). Tables show the number of patients at risk for ICU death per time point after ICU admission. Dashed horizontal line indicates a survival fraction of 0.5. COVID-19, coronavirus disease.
